# A systematic review of methods to measure menstrual blood loss

**DOI:** 10.1186/s12905-018-0627-8

**Published:** 2018-08-22

**Authors:** Julia L. Magnay, Shaughn O’Brien, Christoph Gerlinger, Christian Seitz

**Affiliations:** 1 Institute for Science and Technology in Medicine, Guy Hilton Research Centre, Hartshill, Stoke-on-Trent, UK; 2 Department of Obstetrics & Gynaecology, Keele University School of Medicine, Stoke-on-Trent, UK; 3 Bayer AG, 13342 Berlin, Germany; 4 Gynecology, Obstetrics and Reproductive Medicine, University of Saarland Medical School, Homburg/Saar, Germany

**Keywords:** Alkaline hematin, Alkaline haematin, Heavy menstrual bleeding, HMB, Menorrhagia, Menstrual blood loss, MBL, Menstrual pictogram, PBAC, Pictorial blood loss assessment chart

## Abstract

**Background:**

Since the publication over 50 years ago of the alkaline hematin method for quantifying menstrual blood loss (MBL) many new approaches have been developed to assess MBL. The aim of this systematic review is to determine for methods of measuring MBL: ability to distinguish between normal and heavy menstrual bleeding (HMB); practicalities and limitations in the research setting; and suitability for diagnosing HMB in routine clinical practice.

**Methods:**

Embase®™, MEDLINE®, and ClinicalTrials.gov were screened for studies on the development/validation of MBL assessment methods in women with self-perceived HMB, actual HMB or uterine fibroids, or patients undergoing treatment for HMB. Studies using simulated menstrual fluid and those that included women with normal MBL as controls were also eligible for inclusion. Extracted data included study population, results of validation, and advantages/disadvantages of the technique.

**Results:**

Seventy-one studies fulfilled the inclusion criteria. The sensitivity and/or specificity of diagnosing HMB were calculated in 16 studies of methods involving self-perception of MBL (11 pictorial), and in one analysis of the menstrual-fluid-loss (MFL) method; in 13 of these studies the comparator was the gold standard alkaline hematin technique. Sensitivity and specificity values by method were, respectively: MFL model, 89, 98%; pictorial blood loss assessment chart (PBAC), 58–99%, 7.5–89%; menstrual pictogram, 82–96%, 88–94%; models/questionnaires, 59–87%, 62–86%, and complaint of HMB, 74, 74%. The power of methods to identify HMB was also assessed using other analyses such as comparison of average measurements: statistical significance was reported for the PBAC, MFL, subjective complaint, and six questionnaires. In addition, PBAC scores, menstrual pictogram volumes, MFL, pad/tampon count, iron loss, and output from three questionnaires correlated significantly with values from a reference method in at least one study. In general, pictorial methods have been more comprehensively validated than questionnaires and models.

**Conclusions:**

Every method to assess MBL has limitations. Pictorial methods strike a good balance between ease of use and validated accuracy of MBL determination, and could complement assessment of HMB using quality of life (QoL) in the clinical and research setting.

**Trial registration:**

PRISMA registration number: CRD42016032956.

**Electronic supplementary material:**

The online version of this article (10.1186/s12905-018-0627-8) contains supplementary material, which is available to authorized users.

## Background

Heavy menstrual bleeding (HMB; menorrhagia) is defined by the National Institute for Health and Care Excellence (NICE) in the United Kingdom as excessive menstrual blood loss (MBL) that interferes with a woman’s physical, emotional, social, and material quality of life (QoL). Up to 50% of women of reproductive age (18–54 years) can experience HMB [1, 2], which may cause anemia [3], lead to limitations in social, physical, and leisure activities [1, 4], and be associated with increased healthcare resource use and costs [5].

In clinical practice, the impact of HMB on a woman’s QoL is currently used to guide treatment [1]. However, it is acknowledged that self-perception of MBL can be inaccurate [6], and this may result in surgical intervention in women who are distressed by menses that are actually defined as low volume. General health questionnaires have been found to be inappropriate for use in women with HMB [7], and there is an absence of recommendations for HMB-specific QoL tools [1, 8, 9]. Thus, a method is needed to complement QoL assessments of HMB. Evaluation of MBL informs patient choice by providing context and clarity; for example, the finding of MBL within the normal range may reassure a woman with perceived HMB to the extent that she chooses not to seek further treatment [10–13]. Also, determination of MBL can be important when establishing the etiology of cases of anemia, and changes in MBL are often used to evaluate treatment efficacy in clinical trials.

The alkaline hematin technique, which involves chemically measuring the blood content of used sanitary products, is considered the “gold standard” for MBL determination and has traditionally been used to diagnose HMB as a loss of more than 80 mL of blood per cycle [14, 15]. However, as a result of its practical limitations, its use is mainly confined to the research setting. Consequently, many new approaches to measuring MBL have been developed since publication of the alkaline hematin method more than 50 years ago [16–18]. During this time, sanitary wear has evolved from cotton-based sanitary products to superabsorbent-polymer-containing (SAP-c) ultraslim towels containing granules that can absorb many times their own weight in fluid [19] hence there is a requirement to validate any method for measuring MBL with the same types/brands of sanitary product that the method is intended to be used with. A summary of the validity and merits of existing measurement techniques, including details of the specific products for which they are validated, would be of great benefit to healthcare providers, clinical scientists, and policy makers. To this end, we performed a systematic review with the specific aims of determining: (1) the degree to which methods for measuring MBL are validated to distinguish between normal bleeding and HMB, (2) the practicalities and limitations of each method in the research setting, and (3) whether any of the methods could be used in routine clinical practice to diagnose HMB.

## Methods

The systematic review protocol was registered at PROSPERO (https://www.crd.york.ac.uk/PROSPERO) in March, 2016, with the registration number CRD42016032956. Embase®™ and MEDLINE® were searched using Ovid® on 2 March, 2016, and again on 2 November, 2016. The search for articles concerned with validation/development of methods for assessing MBL was based on strategies used to develop the NICE HMB guidelines [1]. Further terms were added to broaden the range of techniques for assessing MBL among retrieved articles. The search terms can be found in Additional file 1. A search was also performed in ClinicalTrials.gov for relevant ongoing and recently completed clinical trials that investigated methods to measure MBL. The search terms are shown in Additional file 2. Following removal of duplicates, retrieved articles were manually screened based on title then abstract.

Articles were selected for inclusion in the study if the main focus was the development or validation of a measure for assessing MBL in women with self-perceived HMB, actual HMB (MBL > 80 mL per cycle), or uterine fibroids, or in women undergoing treatment for HMB. Validation/development studies that used simulated menstrual fluid and those that included women with normal MBL as controls were also eligible for inclusion. Studies investigating only health-related QoL measures or questionnaires were not specifically sought in the original searches to maintain the focus of the review, but were considered for inclusion during screening. To ensure the capture of methods for which published validation data may not yet be available, we included articles that presented novel or modified methods even if validation was not the main focus. To guarantee that original validation studies were included there were no date limits. Similarly, because some early validation studies had only a few participants, no limitations to population size were applied.

Exclusion criteria were studies that only considered an application rather than the development or validation of a technique, those with non-English abstracts, or articles on irrelevant outcome measures or of an inappropriate publication type; for example, preclinical studies and letters. For each included study, the following data were extracted and independently checked: full reference, study type, setting, population, measurement technique, type of validation, statistical output, advantages/disadvantages of the technique (including sensitivity/specificity scores for measuring MBL, discriminatory power for assessing normal versus high MBL, and additional advantages/disadvantages), and any information on the risk of bias. Inter-cycle and internal consistency were also assessed.

## Results

**Fig. 1:**
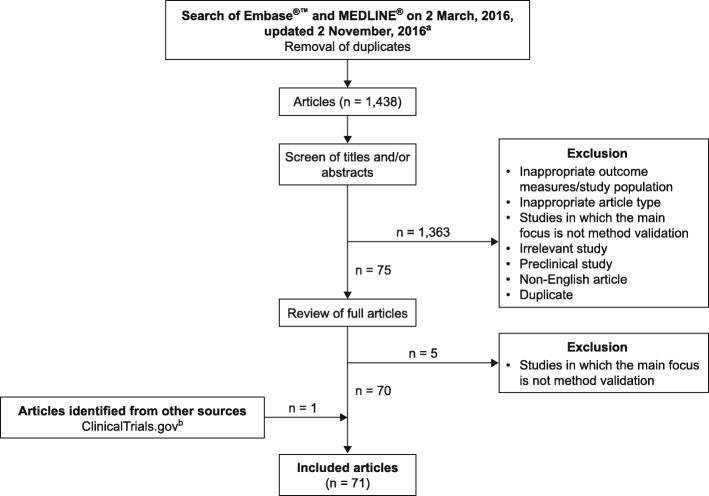
Systematic search and selection process. ^a^See Additional file 1 for search terms. ^b^See Additional file 2 for search terms

On 2 November, 2016, 1438 records were retrieved from Embase®™ and MEDLINE®, including key predefined references [20–35]. Of these articles, 70 fulfilled the inclusion criteria (Fig. 1; Additional file 3) [6, 8, 10, 11, 14, 19–23, 25–84]. Up to 25 January, 2017, 123 relevant entries were retrieved from ClinicalTrials.gov. One trial, NCT01643304, fulfilled the inclusion criteria [85].

Among these studies, population sizes ranged from 2 [14] to 4506 [80]. The risk of bias was rarely formally acknowledged in the included studies, which were also subject to different types of bias. Inter-cycle consistency was assessed in six studies [11, 14, 25, 42, 44, 86], with variable results (Additional file 4). Internal consistency, measured using a variety of methods, was generally good in the 18 studies in which it was considered (Additional file 4). The sensitivity and specificity of diagnosing HMB, or a specified change in MBL, were calculated in 16 studies of tools involving self-perception of MBL (11 pictorial), and in one analysis of the menstrual fluid loss (MFL) method (Table 1). In studies reporting both sensitivity and specificity, the Youden’s statistic, a measure of test performance with an optimal score of 1.0, ranged from 0.33 [20] to 0.88 [26].

**Table 1:** Sensitivity and specificity of methods for measuring MBL

Author(s) and year (reference)	Method	Study population	N (n)^a^	Sensitivity (%)	Specificity (%)	Criteria tested	Concurrent validation method
MFL							
Fraser et al., 2001 [44]	Regression estimation of MBL from total MFL	Women with self-perceived normal or heavy MBL	53 (106)	89 98	98 NR	Diagnosing MBL > 80 mL using MFL cut-offs Diagnosing normal (< 60 mL), heavy (60–100 mL), and excessive (> 100 mL) MBL by predictions based on MFL	AH [14, 40]
PBAC							
Hald & Lieng, 2014 [54]	Modified PBAC (revised icons)	Women with self-perceived light, normal, or heavy MBL	429 (1049)	78.5 20.1–100	75.8 3.1–99.1	Diagnosing heavy MBL with PBAC optimal cut-off of 160 Diagnosing heavy MBL with PBAC cut-offs of 10–450	Subjective assessment of MBL
Zakherah et al., 2011 [33]	PBAC (SAP version)	Women with self-perceived normal or heavy MBL	197 (241)	99 83	39 77	Diagnosing MBL > 80 mL using a PBAC cut-off of 100 Diagnosing MBL > 80 mL using a PBAC cut-off of 150	AH [14, 41]
Reid et al., 2000 [30]	PBAC	Women with self-perceived heavy MBL	103 (103)	97.0	7.5	Diagnosing MBL > 80 mL with a PBAC cut-off of 100	AH [14]
Barr et al.*,* 1999 [20]	Modified PBAC	Nigerian adolescents	281 (562)	58	75	Diagnosing MBL > 80 mL with a PBAC cut-off of 50	AH [14]
Janssen et al., 1995 [25]	Modified PBAC	Women with self-perceived normal or heavy MBL or anemia	288 (489)	91.0 19.1–97.8	81.9 52.3–100	Diagnosing MBL > 80 mL with a PBAC cut-off of 130 Diagnosing MBL > 80 mL with PBAC cut-offs 90–300	AH [14, 35, 40]
Deeny et al., 1994 [22]	PBAC	Women with dysfunctional uterine bleeding	53 (53)	88	52	Diagnosing MBL > 80 mL with a PBAC cut-off of 100	AH [14]
Higham et al., 1990 [23]	PBAC	Women with a range of MBL	28 (55)	86	89	Diagnosing MBL > 80 mL with a PBAC cut-off of 100	AH [14]
Menstrual pictogram							
Magnay et al.*,* 2014 [27]	Menstrual pictogram (SAP version)	Women with self-perceived light, normal, or heavy MBL	119 (235)	82	92	Diagnosing MBL > 80 mL	AH [39]
Larsen et al., 2013 [26]	Menstrual pictogram (excluding extraneous MBL)	Women with confirmed heavy MBL	87 (174)	96	92	Identifying ≥50% decrease in MBL	AH [14, 40]
Wyatt et al., 2002 [59]	Menstrual pictogram Symptometrics device	Women with self-perceived normal or heavy MBL	59 (109)	90	94	Diagnosing MBL ≥80 ml	Paper menstrual pictogram [32]
Wyatt et al., 2001 [32]	Menstrual pictogram (hygiene product icons)	Women with self-perceived normal or heavy MBL	108 (108)	86	88	Diagnosing MBL > 80 mL	AH [14]
Methods involving self-perception of MBL							
Schumacher et al., 2012 [84]	Mixed linear model (menstrual diary and laboratory parameters)	Women with confirmed heavy MBL (≥80 mL during ≥2 reference cycles)	162 (648)	87	70	Diagnosing MBL > 80 ml	AH [14, 40]
Bushnell et al., 2010 [69]	MIQ	Women with self-perceived normal MBL or diagnosed heavy MBL	262 (524)	64 69–79	75 63–82	Predicting meaningful MBL change with MIQ cut-offs of: + 3 from last baseline to first treatment cycle (item 6a) − 0.5 change from baseline (items 1–4)	MIQ item 6c
Lukes et al., 2010 [72]	MIQ	Women with confirmed heavy MBL (≥80 mL in 2 pretreatment cycles)	278 (556)	71.3	61.9	Predicting an optimal MBL reduction of − 22% with a meaningful response on MIQ	AH
Warner et al., 2004 [68]	MEQ (including ferritin status)	Women with self-perceived menstrual complaint	161 (161)	60	86	Diagnosing MBL > 80 mL	AH [14]
Heath et al., 1999 [62]	Menstrual Record Menstrual Recall	Young adult women	29 (29)	Record, 66 Recall, 59	Not mentioned	Correspondence with weighed menstrual loss tertiles (low, normal, high)	Weighed menstrual fluid (MFL)
Janssen et al., 1995 [25]	Subjective assessment of MBL	Women with self-perceived normal or heavy MBL or anemia	288 (489)	74.2	73.9	Diagnosing MBL > 80 mL with complaint of heavy MBL	AH [14, 35, 40]

^a^N = study population size; n = number of cycles studied

*AH* alkaline hematin, *MBL* menstrual blood loss, *MEQ* Menstrual Evaluation Questionnaire, *MFL* menstrual fluid loss, *MIQ* Menorrhagia Impact Questionnaire, *NR* not reported, *PBAC* pictorial blood loss assessment chart, *SAP* superabsorbent polymer

As many women now prefer to use ultraslim towels with enhanced absorbency properties [19], the most relevant methods for measuring MBL in current clinical use and research are those validated for SAP-c products. The validity of measuring MBL with selected SAP-c products was investigated for the alkaline hematin technique, weight assessment of MFL, the pictorial blood loss assessment chart (PBAC), and the menstrual pictogram [19, 27, 33, 38, 39].

### Alkaline hematin method

The original alkaline hematin method was developed for cotton-based sanitary products. It was concurrently validated in one study, showing good agreement with an iron isotope activity method in three phases of one woman’s menstrual cycle (Additional files 3 and 5) [14]. Modified versions of the alkaline hematin method were concurrently validated in two studies. In the first study, mean MBL measured with an automated alkaline hematin method in an Indian population was similar to that determined using a precursor alkaline hematin method in a US population [36, 40, 41]. With the advent of SAP-c sanitary products, the method was revalidated by comparing a semi-automated version for use with a selected brand of SAP-c towels to a manual reference method (*r*^2^ = 0.991; *P* < .0001, *n* = 63) (Additional files 3 and 4) [39]. The power to discriminate between normal bleeding and HMB was assessed for four methods and found to be reasonable, but statistical significance was not given (Additional file 6) [10, 35, 41, 42].

The efficiency of blood extraction from sanitary products using the alkaline hematin method was investigated in 10 studies (Additional file 4) [10, 14, 35–42]. In the original alkaline hematin method, blood recovery was 96.3% after a 20-h incubation [14]. With modifications to improve speed and usability, efficiencies of recovering various volumes of blood from a selection of sanitary products ranged from 74.8% [42] to 107% [41]. Adaptation of the method for a selected brand of SAP-c towels resulted in recovery of at least 90% (≥85% with automation) of simulated menstrual fluid volumes [38, 39].

### Menstrual fluid loss, pad counts, and duration of period

Fluid weight (MFL) has been investigated as a simple alternative to the assessment of MBL by the alkaline hematin method [44]. The measure can be expressed gravimetrically (g) or converted directly to volume (mL), based on the assumption that the specific gravity of menstrual fluid equals one [28, 44]. The relationship between MBL and MFL was considered in four studies, three of which used the alkaline hematin method as the comparator [27, 44, 48]. In all four studies, including one that validated MFL measurements with a selected brand of SAP-c towels [46], there was a correlation between MFL and either MBL or change in MBL (*r* = 0.88–0.97; *P =* .001–.0001; Additional file 5) [27, 44, 46, 48]. In a fifth study, MBL estimated from MFL correlated, but was not interchangeable, with MBL measured by the alkaline hematin method (*r* = 0.73; *P* < .00001; Additional file 5) [84].

In the study by Fraser and colleagues, the sensitivity and specificity of diagnosing HMB with a regression model to estimate MBL from MFL were 89 and 98%, respectively. It was also shown that the blood content in MFL was similar in women with moderately heavy (60–100 mL) and excessive (> 100 mL) MBL (48 and 50%, respectively) [44]. In a later study using SAP-c towels, establishment of a normal range for MFL was prohibited because of overlap of MFL between women with normal and those with excessive MBL, and blood fraction was found to increase progressively with MFL volume [27].

The association of MBL with duration of menstruation was investigated in four studies (Additional files 5 and 6). There was a modest correlation in a study of 207 women complaining of HMB (*r* = 0.35, *P* < .01) [82], whereas no relationship was observed in the other three studies, which included women with a range of MBL volumes [11, 27, 43]. In a study of women with self-perceived HMB, period duration was not significantly different for the lightest versus the heaviest periods [6]. Information on the length of periods was requested in some questionnaires [62, 66], and this parameter forms part of both the PBAC and menstrual pictogram methods of assessing MBL [23, 32].

The association of MBL with counts of sanitary items was considered in eight studies. Higham and Shaw and Warner et al. found that MBL was associated with the total number of pads and tampons used (*r* = 0.61 and 0.30; *P* < .005 and *P* < .001, respectively) [68, 82]. Chimbira et al. found that the median MBL was greater in women using 10 or fewer pads per period than in those using 31–40 pads, but there was a wide scatter of MBL in each group [34]. Five studies found no significant or overall correlation between pad/tampon count and MBL (Additional file 5: Table S5) [6, 27, 32, 42, 82]. A question about the number of pads used by women was included in seven questionnaires [61, 62, 65, 68, 71, 74, 83]; information about the frequency of changing pads was requested in three questionnaires [28, 63, 79].

### Measurement of iron/labelled red blood cells

The discriminatory power of three methods to measure iron/labelled blood in pads [31], from menstrual products [43], or in the whole body [29] was assessed: all of the techniques were able to discriminate between normal and high MBL, or between non-anemic and anemic women, but statistical significance was not reported (Additional file 6). In a fourth study, the amount of iron lost in pads strongly correlated with MFL [47] (Additional file 5).

### Pictorial methods

The validation of pictorial methods was the focus of 19 articles (Additional file 3). These studies evaluated either the PBAC, which uses a scoring system that is proportional but not equivalent to MBL, or the menstrual pictogram, which measures MBL in milliliters and is directly comparable to the alkaline hematin method.

#### Pictorial blood loss assessment chart (PBAC)

Fourteen of the included articles reported on the PBAC/modified PBAC (Additional file 3). In one study, the PBAC was validated for a selected brand of SAP-c products [33]. Sensitivity and specificity of the PBAC were determined in seven studies (Table 1). In six studies these related to diagnosis of a measured MBL > 80 mL [20, 22, 23, 25, 30, 33], and in one study they related to diagnosis of self-perceived HMB [54]. For the diagnosis of MBL > 80 mL, sensitivity was 58–99% and specificity was 7.5–89%. For the diagnosis of self-perceived HMB, sensitivity was 78.5% and specificity was 75.8%. Specificity and sensitivity data generated with a single PBAC cut-off of 100 were presented in three studies [22, 23, 30]. Sensitivity and specificity data derived from a PBAC cut-off of 50 were provided in one study [20]. In three studies, data using multiple cut-off values, including those > 100, were reported [25, 33, 54].

The discriminatory power of PBAC was assessed in nine studies (Additional file 6). Statistically significant results were reported for the difference in PBAC scores between patients in heavy, normal, and light bleeding categories [54, 56], those with and without menstrual disorder [52], individuals before and after treatment [81], and treated patients and active controls [48]. The association of PBAC with MBL (or change in MBL) was assessed in five studies. The range in correlation coefficients across four studies was 0.4659–0.847 (Additional file 5). The generalizability of the PBAC beyond the United Kingdom population of adult women in which it was originally validated was considered in seven studies (Additional file 4). The method was used successfully in populations of adolescents [56, 76], users and non-users of oral contraceptives [55], and Iranian and Turkish women [51, 52, 81]. However, the PBAC may overestimate MBL in the general community [53]. Inter-cycle consistency was assessed with PBAC in two studies and found to be high [25, 54].

#### Menstrual pictogram

**Fig. 2:**
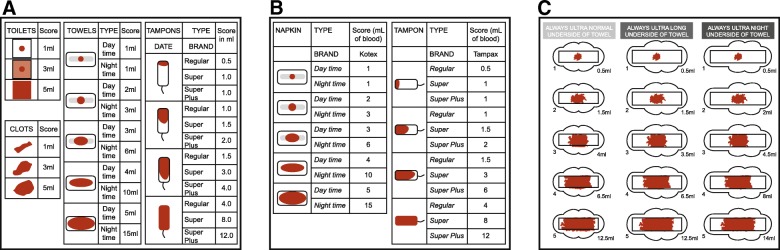
**a** Representation of the original menstrual pictogram. Reprinted from *Fertility and Sterility*, 76, Wyatt KM et al., Determination of total menstrual blood loss, pp125–31, Copyright 2001, with permission from Elsevier [32]. **b** A modified version of the menstrual pictogram. Larsen L et al., *Reproductive Sciences*, 20 (6), pp680–7, Copyright © 2013, Reprinted by permission of SAGE publications [26]. **c** The menstrual pictogram validated for use with towels containing superabsorbent polymers. Reprinted from *Fertility and Sterility*, 101, Magnay, JL et al., Validation of a new menstrual pictogram (superabsorbent polymer-c version) for use with ultraslim towels that contain superabsorbent polymers, pp515–21, Copyright 2014, with permission from Elsevier [27]

The menstrual pictogram/modified menstrual pictogram were the focus of five included articles (Additional file 3). The sensitivity and specificity of the menstrual pictogram were determined in one investigation for a specified decrease in MBL, and in three studies in terms of diagnosing MBL > 80 mL. In one study in which the menstrual pictogram was evaluated as part of the Symptometrics device, the reference method was the paper menstrual pictogram (Table 1) [26, 27, 32, 59]. Across these four studies, the sensitivity was 82–96% and the specificity was 88–94%. A menstrual pictogram specifically designed for use with a particular brand of SAP-c towels (Fig. 2) was endorsed in one of these studies [27]. The predictive power of the menstrual pictogram at diagnosing HMB was presented in one report (positive, 91%; negative, 83%) [26].

Menstrual pictogram/modified menstrual pictogram values correlated highly with MFL assessed by the weight method, and with MBL or change in MBL from baseline assessed by the alkaline hematin method (Additional file 5). The original menstrual pictogram (Fig. 2) was validated in the United Kingdom in untreated women with self-perceived, normal, or heavy MBL [32]. External validation of a modified version of the tool (Fig. 2) was performed in North American women treated for HMB associated with leiomyomata [26] (Additional file 4).

### Self-perception

The specificity and sensitivity of self-perception methods were investigated in six studies, including some questionnaires (Table 1). Complaint of HMB was able to diagnose an MBL > 80 mL with both a specificity and a sensitivity of 74% and a positive predictive value of 56% [25]. A model including diary entries of both self-perceived bleeding intensity and clinical parameters diagnosed MBL > 80 mL with a sensitivity and specificity of 87 and 70% respectively [84]. A questionnaire including a Record and a Recall method for estimating MBL, both of which had a subjective element, resulted in sensitivities of 66 and 59%, respectively. Recall method and Record method tertiles had significantly different mean MFL values [62]. With the Menorrhagia Impact Questionnaire (MIQ), the specificity and sensitivity of changes in MIQ items corresponding to either a meaningful change in perceived MBL [69] or an optimal reduction in MBL measured by the alkaline hematin method [4] were 63–82%. In a study of the Menstrual Evaluation Questionnaire, which includes questions on the self-perceived heaviness of periods, the sensitivity and specificity of diagnosing MBL > 80 mL were 60 and 86% respectively, and women who rated their periods as “very heavy” had a significantly higher mean MBL than the remainder of the women [68].

There was low-to-moderate correlation of 36-Item Short Form Survey (SF-36) score, log discharge rate, ferritin score or PBAC score with scores from four questionnaires: the Menstrual Bleeding Questionnaire [MBQ], the Mansfield–Voda–Jorgensen menstrual bleeding scale, an MBL questionnaire, and a QoL questionnaire (Additional file 5) [8, 28, 73, 83]. All four surveys, as well as the Health Utilities Index questionnaire [75], were able to discriminate between women with and without HMB, with statistical significance attained for the MBQ, the QoL questionnaire, and the Health Utilities Index (Additional file 6). The Portuguese SF-36 did not correlate with PBAC scores [70].

A questionnaire was used in 1547 women to self-grade MBL heaviness and assess the effects of self-perceived HMB on daily life and the Swedish SF-36 [85]. The daily lives of women with self-perceived HMB were affected much more than those of women with normal MBL (Additional file 6). Women with self-perceived HMB also had significantly worse health-related QoL in all domains of the Swedish SF-36 compared with women with normal menstrual bleeding.

The percentage of women with self-perceived HMB and a measured MBL of 80 mL or more was assessed in nine studies and found to range from 26 to 76% [6, 11, 27, 30, 32, 34, 35, 68, 82] (Additional file 6). For three methods involving self-perception of MBL, positive establishment of face and content validity was reported (Additional file 3) [57, 71, 78].

### Additional advantages and disadvantages

The additional advantages and disadvantages of methods for measuring MBL are summarized in Table 2. A more detailed overview is provided in Additional file 3.

**Table 2:** Additional advantages and disadvantages of methods to measure menstrual blood loss

Method	Advantages	Disadvantages
Alkaline hematin	• “Gold standard” in terms of accuracy • Has undergone considerable development to improve rapidity; validated for selected SAP-c towels • Well suited to research setting • Best used in conjunction with a pictorial method and a diary	• Requires calibration curves for each product and does not take extraneous blood loss into account • Patients in the clinical setting may be deterred by having to collect, store, and send sanitary products for analysis
Gynaeseal/Mooncup	• Requires few changes per day	• Subject to leakage during collection and therefore unsuitable for either clinical or research purposes
MFL	• Simple • Can be used to measure effect of medical intervention	• Requires collection of used sanitary products and pre- and post-use weighing • Sanitary item must be stored in a sealed container before weighing to minimize fluid evaporation
Duration of period	• Simple and easy	• Participants must record/recall results
Counts of pads	• Simple and easy	• Participants must record/recall results • Frequency of changing pads can be influenced by many variables
Measurement of iron/labelled red blood cells	• Most methods are accurate	• Technically challenging to perform, requires specialist equipment, and is therefore most suited to research
PBAC	• Quick and simple • Has undergone extensive development; validated for selected SAP-c towels • Suitable for research purposes and has potential value in the clinic	• Only validated for a limited number of current products • Participants must record/recall results
Menstrual pictogram	• Quick and easy • Has undergone development; validated for selected SAP-c towels • Suitable for research purposes and has potential value in the clinic • Differentiates between absorbency ratings of sanitary items	• Only validated for a limited number of current products • Participants must record/recall results
Question-naires	• Many available, ranging in complexity, with questions relating to MBL, generic or disease-specific QoL, or both	• Poorly validated, with a few exceptions • Participants must record/recall results
Self-perception	• Simple • Useful for clinical assessments	• Does not give precise MBL measure • Participants must record/recall results • Individuals can be poor judges of MBL • Not diagnostic

*MBL* menstrual blood loss, *MFL* menstrual fluid loss, *PBAC* pictorial blood loss assessment chart, *QoL* quality of life, *SAP-c* superabsorbent polymer-containing

## Discussion

We present the results of a systematic review of the development and validation of methods for measuring MBL. We summarize the data available and list other key advantages and disadvantages of the various procedures.

The alkaline hematin method has been well validated in terms of the efficiency of blood recovery from sanitary items, including towels that contain superabsorbent polymers as the absorptive agent. It is widely recognized as the most objective technique with which to measure MBL, and can be required by regulatory bodies to assess the efficacy of new treatments for HMB. However, to be suitable for the clinical setting, a test must be quick, easy to conduct, and accepted by patients. In this respect, the alkaline hematin method is less appropriate for use in the general population than the PBAC, menstrual pictogram, or a questionnaire, especially the electronic versions. Despite extensive development to make it more practical and widely applicable, the original inherent disadvantages of the method remain; namely, the need to collect, store and then send all used sanitary items to a laboratory for analysis. In addition, the method is limited to documentation of MBL collected on sanitary items. To overcome this drawback it would be necessary to either carefully capture all extraneous blood loss or use a simultaneous diary to take extraneous blood loss into account. Nonetheless, the alkaline hematin method is widely recognized to be the “gold standard” in terms of accuracy. Ideally, all methods to measure MBL would be validated against alkaline hematin. However, this can be challenging in practice, in part due to the abovementioned disadvantages of the alkaline hematin technique.

Correlations were observed between MFL and MBL, and it is argued that MFL matters more to women concerned with flooding than MBL [48]. However, MBL estimated using MFL is not interchangeable with MBL measured using the alkaline hematin method [84]. Furthermore, variation in the proportion of the non-blood fraction, particularly at extremes of menstrual loss, limits the validity of using MFL to diagnose HMB [27, 87, 88]. Therefore, we do not consider MFL to be a reliable validator of MBL. The MFL method also requires women to store and submit carefully all used products for analysis, which may not be acceptable or feasible, resulting in reduced compliance. In principle, the use of menstrual cups to collect large volumes of MFL is simple. In reality, many women report that spillage and leakage is common and the technique has thus proved unsuitable for measuring MBL.

The hypothesis that menstrual duration alone can be used to predict HMB is not supported by current evidence. It is also not possible to accurately correlate MBL with the number of feminine items used during a menstrual period, and extreme examples have been cited of women with HMB using far fewer towels than those with light menstrual flow [6, 27, 82]. In addition to the brand and absorbency of the product, many variables affect the frequency with which items are changed, including rate and composition of menstrual flow, individual anatomy, ambient humidity, physical activity, posture, and personal fastidiousness in changing sanitary garments. Some patient-reported outcome instruments have attempted to incorporate product absorbencies, albeit that towel absorbency ratings are arbitrary and unregulated [28, 62, 71, 83]. However, the degree of saturation and/or brands of products are not always taken into account [28, 71, 83].

In general, the discriminatory power and sensitivity/specificity scores are reasonably high for the PBAC/modified PBAC, but because some low scores have been recorded the robustness of this test has been queried [20, 25, 30, 89]. The PBAC is not as accurate as the alkaline hematin method for determination of HMB, but it is nevertheless superior to using clinical history or a simple bleeding diary. Moreover, it has been validated for use with selected SAP-c products, does not require laboratory facilities, and has undergone much external validation. The menstrual pictogram has consistently high (> 80%) sensitivity and specificity in diagnosing HMB, including when validated with a SAP-c product. Furthermore, it correlates well with the alkaline hematin method [26, 27, 32, 59], although only five menstrual pictogram validation studies were retrieved. In contrast to the PBAC, the original menstrual pictogram includes a greater range of icons and differentiates between absorbency ratings of sanitary items [32].

Self-assessment of MBL yields a number of false negatives and positives when compared with more objective measurements. The concept that some women have a distorted perception of their MBL is corroborated by data from a 16-study meta-analysis [90]. Among measures of MBL involving self-perception, a mixed linear model based on a menstrual diary and laboratory parameters had the highest sensitivity score (87%), but to date the model has only been tested in a single trial in women with HMB.

Various articles on questionnaires were reviewed. All the questionnaires contained items related to self-perception of MBL or its impact on QoL. Concurrent validation was performed in fewer than half of the questionnaires included, with mixed results [8, 28, 62, 69, 70, 72, 73, 75, 76, 85, 86], but three questionnaires showed promise in terms of ability to discriminate between heavy and normal MBL [8, 73, 75].

Importantly, of all the methods reviewed, only the alkaline hematin method, the PBAC, and the menstrual pictogram are validated for measuring MBL with selected SAP-c products [19, 27, 33, 38, 39]. In addition to the statistical analyses described above, treatment-induced changes in MBL that were also detected by a reference technique were reported for four methods: the menstrual pictogram, PBAC, MFL, and MIQ [26, 48, 54, 69, 72, 81] (Table 1; Additional files 5 and 6).

MBL is only one aspect of the menstrual experience. Pain, pattern, and predictability of timing can all influence a woman’s perception of her period. QoL is recognized to be an important clinical indicator of the effects of menstruation on women [17, 85, 91]. However, QoL is influenced by many factors other than HMB, such as undernourishment and depression [70]. Given that treatment of HMB may incur significant psychological, physical, and financial costs [28], and – as discussed above – validated, quick, economical, and easy methods for assessing MBL are available, future evaluation of a clinical technique that combines a pictorial method validated for modern sanitary products with a daily (preferably electronic) menstrual diary of specific health-related QoL items would be beneficial.

A limitation of this review is that comprehensive comparisons of the different techniques were limited because of the heterogeneity of validation methods and result formats. The definition of HMB was not the same for all methods and there was a lack of consistency in the comparator employed (Table 1 and Additional file 5). With the PBAC, different cut-off values were used to diagnose HMB. Often the PBAC or menstrual pictogram was not used or evaluated in the way in which it was originally validated [50–52, 54], and none of the amendments has been recertified by the alkaline hematin assay. The risk of bias was seldom formally acknowledged in the articles reviewed, and different types of study were subject to different types of bias. Blinding of investigators/gynecologists to participant data was acknowledged in all studies in which investigator–participant agreement was assessed (Additional file 4) [19, 23, 25, 27, 39, 48, 59].

Our searches identified articles that were excluded because they primarily described the application of an existing method for measuring MBL rather than any form of method validation [92–97]. A recent systematic review analyzing the frequency of use of MBL measurement tools in randomized controlled trials found that PBAC score was the most commonly used primary outcome [16]. The menstrual pictogram did not feature, perhaps because it has been used less frequently than the PBAC, but in our systematic review four non-validation studies were identified in which the menstrual pictogram assessed MBL [92, 93, 95, 97]. As a result of the search terms used in our review there may have been incomplete retrieval of reports relating to questionnaires and QoL studies [7, 98–103]. It would be well beyond the scope of this review to expand the existing search strategy to identify all questionnaires relating to MBL. However, it should be considered for a follow-up analysis because there is a need to standardize the validation of questionnaires and QoL tools for measuring MBL [9, 104].

Despite these limitations, we hope that by summarizing all of the available data on the different methods together this review will inform researchers evaluating new techniques of the standard types of validation required. This in turn should help policy makers conduct a robust appraisal of available methods for measuring MBL.

## Conclusions

Every available method to assess MBL has limitations. Pictorial methods strike a good balance between ease of use and validated accuracy of MBL determination; of these methods, the menstrual pictogram has several advantages, not least that it considers different absorbency levels of sanitary items and has an output of MBL volume in milliliters. Currently, clinicians usually base their diagnosis of HMB on a patient’s reported QoL. However, there are drawbacks, particularly in research trials, to relying entirely on health-related QoL devices, which are essentially subjective measures of HMB. A compromise would be to consider MBL *alongside* QoL when deciding how to diagnose HMB and assess effectiveness of treatments.

## Additional files

Additional file 1: **Table S1.** Full electronic search strategy of Embase®™ and Medline. (PDF 44 kb)

Additional file 2: **Table S2.** Advanced search of the ClinicalTrials.gov website. (PDF 32 kb)

Additional file 3: **Table S3.** Overview of types of validation performed, practicalities, and limitations of methods. (PDF 232 kb)

Additional file 4: **Table S4.** Further validation of methods. (PDF 171 kb)

Additional file 5: **Table S5.** Correlations of methods with established standards. (PDF 168 kb)

Additional file 6: **Table S6.** Assessment of discriminatory power of methods for assessing MBL. (PDF 148 kb)

Additional file 7: Completed PRISMA (Preferred Reporting Items for Systematic reviews and Meta-Analyses) checklist. (PDF 133 kb)

## Abbreviations

HMB: Heavy menstrual bleeding
MBL: Menstrual blood loss
MBQ: Menstrual bleeding questionnaire
MFL: Menstrual fluid loss
MIQ: Menorrhagia impact questionnaire
NICE: National Institute for Health and Care Excellence
PBAC: Pictorial blood loss assessment chart; superabsorbent-polymer-containing
QoL: Quality of life
SAP-c: 36-item short form survey, SF-36
