# Supplementary material for: A systematic review of methods to measure menstrual blood loss
Source: BMC Womens Health. 2018 Aug 22;18:142. doi: 10.1186/s12905-018-0627-8 (PMC6106944; doi:10.1186/s12905-018-0627-8)
Supplement: Supplementary file 1 — Table S1. Full electronic search strategy of Embase®™ and Medline. (PDF 44 kb) [file 12905_2018_627_MOESM1_ESM.pdf]

A systematic review of methods to measure menstrual blood loss

SUPPLEMENTAL TABLE 1

Full electronic search strategy of Embase®™ and Medline®, including limits, performed in Ovid® on 2 March, 2016.

Search string	Search term	Number of hits
1	MENORRHAGIA/	12,374
2	HYPERMENORRHEA/	7,039
3	(menorrhag$ or hypermenorrh$).tw.	8,137
4	menometrorrhag$.tw.	752
5	metromenorrhag$.tw.	25
6	(menstru$ adj3 (bleed$ or blood loss)).tw.	6,338
7	(heavy adj1 (period$ or menses or menstrual or menstruation)).tw.	2,157
8	(dysfunction$ adj3 (uterine or uterus) adj3 (bleed$ or blood$)).tw.	1,984
9	or/1-8	21,121
10	SELF REPORT/	113,004
11	self assess$.tw.	28,162
12	pbac.tw	785
13	pictorial blood loss.tw.	226
14	pictorial chart$.tw.	69
15	pictogram$.tw.	670
16	electronic medical record/ or electronics/ or recording/	166,260
17	radiochromium.tw.	335
18	BLOOD VOLUME/	52,948
19	(paper adj3 scale$).tw.	1,348
20	visual analog$.tw.	105,153
21	((subjective$ or objective$) adj1 (assess$ or test$)).ti.	4,009
22	(menstru$ blood adj1 (loss or volume) adj3 (estimat$ or measur$ or determin$ or assess$)).tw.	400
23	menstrual cup/	25
24	menstrual cup.tw.	50
25	incontinence pad$.tw.	432
26	alkaline h?ematin.tw.	224
27	(indirect methods or ((menstrual or fluid) adj1 weight) or fluid volume or ((menstrual or iron) adj1 loss) or Fe or iron or ferrous or h?emoglobin or radioactive$ or radiolabel$ or counts or tracer).tw.	1,252,218
28	(colo?rimetr$ or atomic absorption or absorptiometry).tw.	133,969
29	or/10–28	1,827,834
30	and/9,29	2,751
31	conference abstract.af.	2,368,161
32	30 not 31	2,299
33	Remove duplicates	1,458

Lines 1–8 were included to locate research related to the health condition. Lines 10–28 were included to locate research related to methods for measuring MBL. The articles retrieved were then screened for studies with an English language abstract on the development or validation of a measure for assessing MBL in women with self-perceived HMB, actual HMB (MBL > 80 mL per cycle), or uterine fibroids, or in women undergoing treatment for HMB. Validation/development studies that used simulated menstrual fluid and those that included women with normal MBL as controls were also eligible for inclusion.

$ = wildcard search term; ? = optional wildcard search term; adj = specifies that the search terms appear within a specified number of words of each other; af = specifies that the search will include all fields; ti = restricts the search to title; tw = limits the search to text words.
