# Supplementary material for: A systematic review of methods to measure menstrual blood loss
Source: BMC Womens Health. 2018 Aug 22;18:142. doi: 10.1186/s12905-018-0627-8 (PMC6106944; doi:10.1186/s12905-018-0627-8)
Supplement: Supplementary file 2 — Table S2. Advanced search of the ClinicalTrials.gov website. (PDF 32 kb) [file 12905_2018_627_MOESM2_ESM.pdf]

A systematic review of methods to measure menstrual blood loss

SUPPLEMENTAL TABLE 2

Advanced search of the ClinicalTrials.gov website performed on 2 March, 2016, and updated on 25 January, 2017.

**Category**	**Terms used**
Search terms	uterine fibroids OR menorrhagia
Conditions	uterine fibroids OR menorrhagia
Outcome measures	Bleeding
