# Supplementary material for: A systematic review of methods to measure menstrual blood loss
Source: BMC Womens Health. 2018 Aug 22;18:142. doi: 10.1186/s12905-018-0627-8 (PMC6106944; doi:10.1186/s12905-018-0627-8)
Supplement: Supplementary file 3 — Table S3. Overview of types of validation performed, practicalities, and limitations of methods. (PDF 232 kb) [file 12905_2018_627_MOESM3_ESM.pdf]

A systematic review of methods to measure menstrual blood loss

SUPPLEMENTAL TABLE 3

Overview of types of validation performed, practicalities, and limitations of methods.

Study (reference)	Concurrent validation (reference)	Other validation	Advantages	Disadvantages
AH				
Original AH method [1]	• Iron isotope activity	• Recovery of blood from sanitary pads and tampons	• Simple, accurate, convenient, no complaints of odor (samples sent by mail)<br>• Towels and tampons can be combined in assay<br>• Well established as a clinical assay and in the research setting	• Towel/tampon color can affect assay
Original AH method (increased incubation time) [2]		• Discrimination of HMB<br>• Recovery of blood from towels and tampons (Modess®, Kotex®), tampons (Tampax®), or cotton sponges (Rondic®)<br>• Internal consistency	• Reliable and simple<br>• Good accuracy down to 0.1 mL<br>• Satisfactory recovery of blood can be achieved with different brands/types of sanitary product<br>• Participants using their customary method of sanitary protection are more likely to cooperate<br>• Soiled products may be stored for ≤1 month	• It is not known if storage of soiled products for >1 month results in hemoglobin degradation<br>• The reason for poorer blood recovery from one brand (Kotex® towels) tested is unknown
Modified AH method (Stomacher® blender, automation) [3]		• Recovery of blood from towels (Kotex® Simplicity low bulk, Dr White's® Maternity high bulk, Kotex® New Freedom, Libresse® Comfort, Boots® Femina, Nikini®, Lilia®) and tampons (Tampax® Regular, Tampax® Super, Lil-Lets® Regular, Lil-Lets® Super, Lil-Lets® Super-plus)<br>• Internal consistency	• Rapid (15 min for stable eluate)<br>• Many blood samples can be analyzed at low cost and within a short period of time<br>• Minimizes exposure of technicians to discomfort<br>• Suitable for routine laboratory use, even in the tropics	• Towel/tampon color can affect assay

Study (reference)	Concurrent validation (reference)	Other validation	Advantages	Disadvantages
Modified AH method (Stomacher® blender, automation) [4]	• AH method [2]	• Recovery of blood from four towels (Skylark®, Carefree®, Comfit®, and Stayfree®)<br>• External validation (Indian sanitary pads)<br>• Intracycle consistency	• Quick (15 min per extraction)<br>• Practical for use in India with various sanitary pads<br>• Can be used to assess the effect of sterilization, intrauterine devices, and oral contraceptives on MBL	
Modified AH method (Stomacher® blender, and modified equation for calculation of MBL including a mean "blank" absorbency for different brands of sanitary products) [5]		• Recovery of blood from towels (Kotex® Maxi) and tampons (Tampax® Super)<br>• Analysis of blanks<br>• Internal consistency<br>• Validation of discriminatory power	• Accurate, convenient, minimal odor (fume hood)<br>• Avoids manual squeezing of sanitary wear and shortens the AH procedure<br>• All patients agreed that it was "not too much bother"<br>• Assessors agreed that the measurement was "not more unpleasant to perform than many other routine laboratory procedures in urine or feces"<br>• Can be performed as a routine laboratory procedure and is suitable for clinical use	• Blank values of each type of towel or tampon must be determined and used in the calculation of MBL
Modified AH method (volumetric test; non-caustic chemicals used for extraction of hemoglobin; standard curves produced) [6]		• Recovery of blood from towels (Stayfree® Maxi Regular or Thin and Mini) and tampons (Tampax® or OB®)<br>• Validation of discriminatory power	• Better than long, cumbersome methods using radioisotopes<br>• Simple, rapid (2 h for extraction and measurement), accurate, reliable, no unpleasant odors, only small volumes of caustic solution<br>• Can aid the diagnosis and evaluation of treatment of dysfunctional uterine bleeding	• Extraction efficiency varies with product (standard curves required for each product used)<br>• With high MBL, samples need to be diluted for measurement
Modified AH method (detergent extraction; photometric analysis; calibration factor applied for specific brands of feminine products) [7]		• Recovery of blood from towels (Kotex® Simplicity Super) and tampons (Tampax® Super Plus)<br>• Internal consistency<br>• Validation of discriminatory power	• Rapid extraction (<30 min) of a complete collection of sanitary material<br>• Clear, simple instructions (89% of women collected products)<br>• A single measurement is sufficient for diagnosis of HMB (a second MBL measurement can be used in cases of obvious methodological problems)	• High cooperation rates might not be achieved outside a research study
Modified AH method (Stomacher® blender and absorbance at A_{564} to measure hemoglobin instead of AH; combined with menstrual diary)		• Recovery of blood from towels (Libresse® Good Night and Invisible Super) and tampons (OB® Fleur Plus and Extra Plus)	• A measurement kit with detailed instructions, sanitary protection, and storage bags resulted in a 99% acceptance rate, but this could also be because of the clinical trial setting and the inclusion of only women with HMB<br>• Combining a diary with a pictorial chart test (and this AH test) would give the best accuracy	• AH test alone does not measure all fluid content of the menstrual discharge<br>• AH test gives no details of bleeding pattern

Study (reference)	Concurrent validation (reference)	Other validation	Advantages	Disadvantages
[8]				
Modified AH method (for SAP-c towels) [9]		• Recovery of a range of simulated menstrual fluid volumes from SAP-c towels (Always Ultra with Wings Normal, Long, Night)	• Accurate, minimal odor<br>• Samples may be stored for 3 weeks at all tested temperatures without loss of recovery<br>• Towels can be processed individually/in batches<br>• Does not need specialized laboratory facilities<br>• Can be readily used in the clinical research setting	
Modified AH method (rapid AH technique for SAP-c towels) [10]	• A manual reference method for blood extraction from towels	• Recovery of a range of simulated menstrual fluid volumes from SAP-c towels (Always Ultra with Wings Normal, Long, Night)	• Reliable, accurate<br>• Rapid (a Stomacher® could process 16 towels/h)<br>• Reduced handling of caustic sodium hydroxide<br>• Direct measurement of hemoglobin levels<br>• Samples could be stored for 3 weeks at 4°C without loss of recovery<br>• Can be readily used in the clinical research setting	• Conversion factors are required for each product used
MFL and menstrual cups				
MFL [11]	• AH method [3]<br>• PBAC	• Validation of discriminatory power<br>• Validated for towels (Kotex® Simplicity 2) and tampons (Tampax® Super)	• MFL is possibly of more relevance than MBL to women (who may be more concerned about flooding than whether discharge is blood or fluid)<br>• Strong correlation between change in total MFL and MBL suggests that total MFL measurement could be used as an assessment of HMB, while being much easier to perform	
Regression estimation of MBL from MFL [12]	• AH method [1,3]	• Inter-cycle consistency<br>• Validated for regular or super towels without wings and tampons	• Provides an accurate estimate of MBL in women with moderate to very heavy losses and appears to be more accurate than PBAC<br>• MBL classification can be made from the measured total MFL directly without having to calculate blood loss<br>• Self-sealing plastic bags for storage eliminated odor<br>• Total MFL is much easier to ascertain than MBL, only requiring weighing of sanitary products before and after use, and a commercial pack for this purpose would be inexpensive and simple<br>• The tool is not subject to behavioral distortion, as was the number of menstrual hygiene products needed, or perceptual bias<br>• For most clinical purposes, the estimate of MBL could be made ignoring the hygiene product type (sanitary pad or tampon)	• Women have to collect all menstrual loss meticulously including "clots"
MFL		• Validation of	• MFL may be a simple alternative to PBAC and the AH	• Overlap of MFL data existed

Study (reference)	Concurrent validation (reference)	Other validation	Advantages	Disadvantages
(for SAP-c towels) [13]		discriminatory power<br>• Validated for Always Ultra towels<br>• External validation	method in women without urinary incontinence<br>• Can be used to measure the effect of medical intervention on MFL	between bleeding categories
Gynaeseal [14]	• AH method [1]		• 95% of women found Gynaeseal easy to insert; one required assistance in removing the Gynaeseal; one parous women was unable to insert the device<br>• 60% of women with normal MBL required no supplementary sanitary wear<br>• "Change the Gynaeseal only once per day" (women with normal loss, n = 6)<br>• "Ability to have sexual intercourse during menstruation" (n = 3)	• All women with HMB who tried to aspirate menstrual fluid from the lower chamber of the Gynaeseal reported spillage of menses<br>• Only five of 22 women submitted written measurements of menstrual volume<br>• All women with HMB said they preferred the standard method of menstrual fluid collection because menstrual spillage was unpleasant<br>• Compared with standard sanitary wear: the seal was "messy to remove" (n = 16; 73%); the odor of the latex was "offensive" (n = 2); and the device was not disposable by lavatory flush (n = 5)
Mooncup [15]	• Sanitary pads and tampons		• Mooncup was changed, on average, less frequently than tampons AND pads<br>• From follow-up questionnaire: "less leakage during activity"	• Greater reported frequency of leakage compared with pads AND tampons<br>• It was changed, on average, 2.3 times more frequently than pads<br>• From questionnaire: "difficult to remove"; "painful"; "uncomfortable on insertion and when in situ"; "frequent emptying and leakage"; "unhappy having to clean it in the kitchen" (hygiene reasons); "having to change the Mooncup in public toilets where facilities are limited"

Study (reference)	Concurrent validation (reference)	Other validation	Advantages	Disadvantages
				• High dropout rate
Measurement of iron/labelled red blood cells				
Radioactive iron (Fe^{59}) [16]		• Validation of discriminatory power<br>• Internal consistency<br>• Validated for "sanitary napkins" and Tampax® tampons	• Highly accurate	• Requires collection of soiled radioactive products
Radioactivity counting dome (Cr^{51}) [17]		• Validation of discriminatory power<br>• Accuracy<br>• Validated for bespoke pads	• Simple, accurate technique<br>• Eliminates count rate errors due to geometric irregularity of the sample	• Quite technical<br>• Requires specialist bespoke equipment<br>• Patients had to use bespoke pads throughout the study and have blood labelled with Cr^{51}
Fe^{59} whole body counting [18]		• Validation of discriminatory power	• Relatively simple<br>• No need for collection of menses or pads<br>• Of great value for routine clinical investigation of anemias where HMB or some other form of blood loss is suspected	• Not as accurate as some techniques (an error of 20–40 mL per period may not be unusual)
Atomic and chemical analysis of iron recovery [19]	• Chemical analysis<br>• Atomic analysis	• Reproducibility<br>• Validated for unspecified pads and tampons	• Estimation of menstrual iron losses are similar for both methods but atomic absorption avoids the necessity of preparing special glassware, is quicker, and helps to reduce errors that may occur during dilution<br>• Does not use derivatives of hemoglobin which may be destroyed by bacterial action, or colorimetric reactions that might suffer from interference by contaminants	• Final estimates of iron may differ by as much as 6 mg between techniques
Menstrual iron loss [20]	• MFL	• Validated for unspecified pads and tampons	• The first time iron losses have been directly measured from menstrual fluid<br>• Can be used to ascertain abnormal MBL before iron-deficient erythropoiesis has developed	
PBAC				
Original PBAC method [21]	• AH method [1]	• Agreement between participant and investigator<br>• Validated for Tampax®/Kotex® Fems Super Plus tampons; Kotex® Simplicity 2 towels	• Simple, reasonably accurate<br>• In general, patients found the charts easy to use<br>• Superior to using counts of towels/tampons (not biased by extreme cases of towel/tampon use)<br>• Could be of value to both clinical practitioners and research workers, especially if it is not possible to measure MBL objectively	• Not as accurate as AH method

Study (reference)	Concurrent validation (reference)	Other validation	Advantages	Disadvantages
PBAC<br>[22]	• AH method [1]	• Validated for customary sanitary wear	• Suitable for use in women complaining of heavy MBL; however, it should not replace the use of the AH method as the “gold standard”	• Discriminatory power is only intermediate for detecting heavy MBL
Modified PBAC (modified icons)<br>[23]	• AH method [1,3,5]	• Agreement between participant and investigator<br>• Validated for Kotex® Maxi Long pads; Tampax® Super tampons	• Superior to subjective assessment of MBL at predicting HMB, even if performed only once<br>• None of the participants found the chart too difficult to complete<br>• No need to collect sanitary wear<br>• Can be used to make therapy more adequate and rational	• Does not give a measure of MBL in milliliters
PBAC<br>[24]		• External validation (general community)<br>• Validated for unspecified pads and tampons with same absorbency as original study		• PBAC may overestimate MBL in the general community
Modified PBAC (revised icons and rating system)<br>[25]		• Validated for unspecified pads and tampons	• Appropriate for measuring any apparent change in MBL	
Modified PBAC (simplified, but with additional questions)<br>[26]	• AH method [1]	• Validated for unspecified products	• Probably the best available tool to health workers in developing countries for managing abnormal blood loss in girls	• Required simplification for a poorly educated population<br>• Should be used with a dysmenorrhoea questionnaire
PBAC<br>[27]	• AH method (reference not given)	• Validated for Tampax® Super;<br>Kotex® Simplicity 2	• Simple	• Inaccurate and therefore does not appear to be of clinical value
PBAC<br>[11]	• AH method [3]<br>• MFL	• Validation of discriminatory power<br>• Validated for Tampax® Super;<br>Kotex® Simplicity 2	• Easy to use	• Care should be taken in interpreting PBAC scores (relationship between PBAC and MBL/MFL is not direct)
PBAC<br>[28]		• External validation (Turkish women)<br>• Validation of discriminatory power<br>• Validated for	• Simple, non-laboratory method, semi-objective<br>• Number and size of any clots passed are taken into account and scored<br>• PBAC is recommended for the diagnosis of HMB and evaluation of treatment outcomes	• Validity of PBAC has been debated<br>• Not gold standard

Study (reference)	Concurrent validation (reference)	Other validation	Advantages	Disadvantages
		unspecified pads and tampons		
PBAC (for SAP-c towels) [29]	• AH method [1,2]	• Validated for Always Ultra towels	• Simple, accurate<br>• Could be used in clinical practice to aid decisions about treatment and follow-up	
Modified PBAC (revised icons and used to estimate volume loss vs a score) [30]		• External validation (Iranian women)<br>• Discriminatory power<br>• Validated for unspecified products	• All women completed the PBAC	• Subject to recall bias
PBAC [31]		• External validation (adolescents)<br>• Validation of discriminatory power<br>• Validated for unspecified pads and tampons	• Quick (<10 min to complete)	• Subject to recall bias
Modified PBAC (revised icons) [32]	• Subjective assessment of MBL	• Reliability<br>• Validated for women's own choice of sanitary items	• Quicker and less expensive than AH method<br>• Does not require collection of sanitary material<br>• A low PBAC score may be used to define treatment endpoints for clinical use or in trials	• Less accurate than AH method
e-PBAC e-BQ [33]		• External validation (bleeders vs non-bleeders, oral contraceptive users vs non-users)<br>• Validated for unspecified products	• Facilitated collection of menstrual data in real time<br>• Easy and straightforward to complete<br>• Patients prefer electronic methods to paper methods<br>• Electronic methods aid data collection/analysis and potentially reduce the chance of errors resulting from manual calculation of bleeding scores<br>• Combination of e-BQ and e-PBAC to assess or screen bleeding in women in the general female population is recommended	
Menstrual pictogram				
Original menstrual pictogram [34]	• AH method [1]	• Validated for Tampax® Regular, Super, Super Plus; Kotex® Maxi Super, Maxi Night-time towels	• Includes extraneous blood loss (clots and in bowl)<br>• Simple, effective<br>• Provides a semi-quantitative estimate of extraneous blood loss in milliliters<br>• More accurate than the original PBAC, probably owing to a	

Study (reference)	Concurrent validation (reference)	Other validation	Advantages	Disadvantages
			wider range of icons/absorbency levels<br>• Could easily be used in primary and secondary care to diagnose HMB, and thus improve clinical treatment decisions and outcomes<br>• Could be used in studies to determine total objective MBL in a normal population	
Menstrual pictogram as part of a Symptometrics device [35]	• AH method [1]	• Agreement with paper charts<br>• Validated for unspecified sanitary pads	• Fast (mean time to measure, analyze, and transfer to the computer: one set of paper-based data, 118 min; data from the Symptometrics device, 5 min)<br>• 48% of participants preferred the Symptometrics device to the paper equivalent (36%) to collect data<br>• Instant display of symptom data makes diagnosis and management more precise and appropriate<br>• Could revolutionize clinical and research practice of menstrual cycle disorders	
Modified menstrual pictogram (excluding extraneous MBL and revised icons) [36]	• AH method [1,3]	• External validation (North American treated population)<br>• Validation of discriminatory power<br>• Validated for Tampax® Regular, Super, Super Plus; Kotex® Maxi Super, Maxi Night-time towels	• Simple<br>• Avoids need to collect soiled products<br>• Could be useful in clinical practice to determine whether the patient-reported HMB meets the criteria for HMB and to help to decide if surgical intervention is necessary<br>• Can be used in clinical trials to detect endpoints in response to treatments for MBL	• The agreement between menstrual pictogram and AH scores was weaker when there was heavy staining of towels
Modified menstrual pictogram (for SAP-c towels) [37]	• AH method [9]	• Internal consistency<br>• Validated for Always Ultra with Wings Normal, Long, Night	• Women found the pictogram easy and quick to use and no one reported any difficulty in matching towel stains to pictogram icons<br>• Can easily be carried in a normal-sized handbag, although a more robust format is required for routine use<br>• Obviates the need to send towels to a laboratory for assessment<br>• The tool would be suitable for testing in a clinical trial	• Not as accurate as the AH method or soiled towel weight (volume assignment is by five discrete images)<br>• In the clinical setting, feminine towels (and hence stain spread) would be affected by mechanical pressures of the wearer
Modified menstrual pictogram (for SAP-c towels, correcting for change in blood fraction with	• AH method [10]<br>• MFL	• Participant vs investigator agreement<br>• Validated for Always Ultra with Wings Normal, Long, Night	• In general, the menstrual pictogram was easy to use with respect to towel color codes, the scorecard, and icon matching<br>• Storing the towels and using the pictogram under everyday conditions were considered satisfactory<br>• Avoids the inconvenience of collecting used feminine	• Excludes extraneous blood loss<br>• Icon assignment difficult when the towel stain differs from depictions<br>• Individual perception and

Study (reference)	Concurrent validation (reference)	Other validation	Advantages	Disadvantages
volume)<br>[38]			products for laboratory assessment<br>• The scoring environment was not considered to be a problem<br>• A simple means of measuring MBL in the clinical setting<br>• Ideally suited to clinical trials to screen women for HMB and to assess clinical improvement during the investigation of new treatment regimens	interpretation of stain areas or incorrectly recorded data may result in inconsistencies<br>• Assigned scores may be inappropriate for non-validated products
Questionnaire				
Postal questionnaire (grading MBL as light, moderate, heavy, or very heavy)<br>[39]			• High response rate	
Menorrhagia multi-attribute utility assessment scale, (considers practical difficulties, effects on social life, psychological effects, physical health, interruption to work, and effects on family life)<br>[40]		• Face and content validation	• Allows assessment of the impact of both the actual disease process and interventions to alleviate it<br>• Intended to have clinical relevance in that it is designed to allow clinicians to assess a woman's current perception of her health, using a simple to administer clinical scale<br>• Might be used longitudinally to monitor the change in health of a patient during a series of interventions	
Menstrual record (function of reported saturation and absorbency of each product)<br><br>Menstrual recall (function of number of products used, absorbency, and number of heavy/light days)<br>[41]	• MFL	• Validation of discriminatory power<br>• Validated for unspecified sanitary pads and tampons	• Non-invasive; quick to administer and analyze; has a low respondent burden (does not require a high level of participant motivation); takes into account different products used in clinical practice<br>• The Menstrual recall method is preferable in an epidemiological setting to the Menstrual record and MFL methods, and is ideal in population studies requiring discrimination between heavy and normal MBL	• Recall method does not give precise measure of MBL and relies on memory<br>• Record method is time-consuming for the participant and the information is difficult to collect and analyze<br>• Could result in the misclassification of participants' MBL and is not an accurate means of diagnosing HMB in the clinical setting
Menstrual score questionnaire (self-perceived debility			• Semi-objective	• 60.6% response rate

Study (reference)	Concurrent validation (reference)	Other validation	Advantages	Disadvantages
in terms of dysmenorrhoea, heavy days, frequency of changing products, clots, flooding, time off work, and duration of the problem)<br>[42]				
Electronic calendar (self-assessment of bleeding as none, spotting, normal, or heavy)<br>[43]		• Comparison with paper version	• Requires less time for data entry and cleaning of data compared with paper forms<br>• Can include a large amount of information because data entry can be simplified with pull-down screens, icons, and the programming of complicated skip patterns into the software<br>• Error checks are built in<br>• Results of an evaluation survey suggest a general acceptance of the electronic calendar by women	• Data entry has an average lag time of 2 days
Interview to determine flow (number of products/cycle) over the previous 5 years<br>[44]		• Validated for unspecified pads and tampons		• Recall bias<br>• The clinical importance of very small menstrual cycle changes may not represent a substantial public health concern
Questionnaire assessing self-perceived changes in MBL (lighter, same, heavier, more variable, stopped) over 12 months<br>[45]			• Self-reporting of heavier MBL as a marker for menstrual dysfunction is subjective but is in keeping with usual clinical methods	• Respondents were asked about a change in MBL rather than if they actually had HMB<br>• Wording of the questions affects accuracy<br>• Subject to recall bias
General Health Questionnaire and women's perception of the heaviness of MBL questionnaire<br>[46]			• 76% response rate	• Psychological distress may increase the self-reporting of gynecological physical symptoms in a community sample
MVJ<br>(6-point Likert scale based on the frequency of required changes of defined absorbency	• MFL	• Validation of discriminatory power<br>• Validated using 87 different brands of towels/tampons	• Simple and practical<br>• Particularly sensitive when estimating moderate to heavy bleeding (three periods should be assessed)<br>• A practical and inexpensive method for clinicians to use with midlife/perimenopausal patients (group most vulnerable to	• Based on the perception of total MFL<br>• Does not measure MBL (can use to estimate MBL)<br>• Some participants did not

Study (reference)	Concurrent validation (reference)	Other validation	Advantages	Disadvantages
products)<br>[47]			worry and/or interventions when HMB is incorrectly diagnosed)<br>• An MVJ score of 6 may indicate a bleeding volume of some concern	understand how to use the MVJ correctly<br>• Did not work well in "light bleeders"
MEQ<br>(includes questions on subjective heaviness of period, type and number of products used, and clots)<br>[48]	• AH method [1]	• Validation of discriminatory power<br>• Validated for towels (Bodyform® Ultra Super and Super Plus sizes) and tampons (Tampax® Regular and Super)<br>• Inter-cycle consistency	• Validated with real-life data<br>• First study to ask whether the period for which blood was collected was representative of periods as reported<br>• Provides a moderately good prediction of patients with an MBL >80 mL	• More than one cycle should be analyzed
Postal questionnaire on menstrual symptoms (i.e., heaviness, duration, if problematic)<br>[49]		• Focus group discussions to check suitability for women in a community setting	• Missing responses for questionnaire items were never more than 2.3%<br>• Women may be better served by a broader clinical assessment of the impact of periods on QoL and closer attention to the aspects of periods they find problematic	• Does not include all factors that contribute to perception of periods<br>• Exact wording of the questionnaire is important<br>• Response rate of 61.5% (women with HMB are more likely to respond)<br>• Discrepancies in use of the terms "symptom" or "problem" in questionnaires vs in clinical practice
Questionnaire assessing MBL (number of pads used; if physician was consulted)<br>[50]		• Questionnaires were pretested in one school before they were used in the field<br>• External validation (Malaysian adolescents)<br>• Validated for unspecified pads	• May help to meet the need for routine screening for dysmenorrhoea in school health programmes	• Self-reporting<br>• Certain survey questions were not answered (some students may not have understood the questions)<br>• Those who had left school were not surveyed, so the results cannot be generalized to all female adolescents
Questionnaire assessing changes in MBL including number of pads used and QoL<br>[51]		• Validated for unspecified pads		• Relies on subjective perception of reduction in MBL<br>• 6/23 patients did not answer the questionnaire

Study (reference)	Concurrent validation (reference)	Other validation	Advantages	Disadvantages
Free-text questionnaire and follow-up qualitative interviews in patients with HMB [52]		• Face validity (coding schemes checked with two other researchers)<br>• The analysis used constant comparison to check for the presence of each coding theme in respondents' accounts and to seek counter examples	• Free text allowed the collection of responses in women's own terms, without prejudgment<br>• The breadth of data from a large number of women given the questionnaire complemented the greater depth of information from a smaller number of qualitative interviewees<br>• The community setting allowed exploration of concerns about menstrual symptoms among a population whose concerns were previously under-explored; most studies are carried out among patients referred to secondary care	• Categorization of free-text answers was difficult; it was subject to interpretation<br>• Distinction between symptoms on questionnaire vs those reported in consultation<br>• Responders (61.5%) may differ from non-responders (e.g., in motivation to complete the questionnaire or in literacy); this limits the generalizability of the questionnaire<br>• Response bias (more missing free-text data than answers to fixed choice questions)
QoL questionnaire (adapted from adult QoL questionnaires about perceptions, symptoms, and family history of bleeding problems) [53]	• PBAC [21]	• Validation of discriminatory power<br>• External validation (adolescents)		
MIQ (health-related QoL) [54]	• AH method (reference not given)		• Considers the importance and meaningfulness of MBL changes to women	• Further studies are needed to establish a minimally important change in MBL in women with and without HMB
MIQ (health-related QoL) [55]	• MIQ item 6c<br>• AH method (reference not given)	• Construct and convergent validation<br>• Validation of discriminatory power<br>• Test–retest evaluation<br>• Reliability of individual items<br>• Assessment of the respondent burden	• Quick (2 min to complete)<br>• Able to differentiate for each MIQ item the degree of change that women with HMB perceived as being clinically meaningful<br>• Not limited by a proof of robustness vs other analytical tools because it uses "meaningful change"<br>• If the observation period is short, a 1-point positive change in scores may be considered a desirable and easily interpretable outcome<br>• The respondent burden is minimal and appropriate for diagnostic and clinical research settings	

Study (reference)	Concurrent validation (reference)	Other validation	Advantages	Disadvantages
SF-36 (general health status; Portuguese version) [56]	• PBAC<br>• Hemo-globin levels	• External validation (Portuguese population)	• Practical<br>• Can be useful to guide efforts to improve clinical assistance	• The presence of other factors such as poor diet, undernourishment, and depression can be reflected in the physical/mental scores
Electronic daily uterine fibroid symptom diary (patient-reported outcomes in the form of 11 items: five addressing menstrual bleeding or spotting; one each related to cramping [distinct from other pain], fatigue, and bloating; and three addressing other fibroid-related pain) [57]		• Face and content validation	• Concept saturation was achieved, indicating that the eight items contained in the diary were relevant to women with uterine fibroids and consistent with how they viewed their symptoms<br>• Every symptom addressed in the diary was endorsed by at least two-thirds of the participants, resulting in a succinct questionnaire appropriate for daily administration<br>• Average time for completing the full diary was 1–2 min per day<br>• Most participants reported that the questions were ‘‘straightforward,’’ ‘‘applicable,’’ and captured the most bothersome symptoms associated with their uterine fibroids<br>• Participants understood how to answer the diary questions and most participants stated that they could distinguish fibroid-related symptoms<br>• It is expected that total MBL can be estimated as a function of the daily ratings on the bleeding item, the presence of clots, and sanitary product use	• The diary is not ideal for evaluating symptom changes or treatment response in the small minority of women with uterine fibroids who have no bleeding symptoms<br>• Owing to the size of the screen of the handheld device, and because it was felt that participants could report the number of saturated sanitary products used more reliably than estimating the portion of saturation on the basis of a picture, the PBAC was not incorporated<br>• Diagnostic utility, as well as known groups, criterion and concurrent validity require assessment
Hospital for Sick Children bleeding questionnaire (question on heaviness of bleeding during period) and PBAC [58]	• Subjective assessment (initial questionnaire)	• External validation (adolescents)	• A combined PBAC and Hospital for Sick Children bleeding questionnaire approach detected more cases of HMB in adolescents than a single question<br>• Development of screening programs for HMB in adolescents in schools using both a bleeding questionnaire and a PBAC would improve detection and treatment of HMB	• High dropout rate<br>• Difficult to perform research on HMB in adolescents
Health Utilities Index questionnaire (assesses health-related QoL based on eight attributes: vision, hearing, speech, ambulation, dexterity, emotion, cognition, and	• Clinical History Assessment Tool	• Validation of discriminatory power<br>• External validation (women with von Willebrand disease)	• Differences in mean utility scores ≥ 0.03 for health-related QoL, and ≥ 0.05 for single attributes, are considered to be clinically important	

Study (reference)	Concurrent validation (reference)	Other validation	Advantages	Disadvantages
pain)<br>[59]				
Web-based questionnaire including disease-specific questions, on the perceptions of MBL<br>[60]	• SF-36<br>• Quality of daily life		• Accurately predicted the prevalence of HMB<br>• Response rate was sufficient for power requirements	• 4% of returned questionnaires were excluded because the question about perception of MBL was not answered
Postal questionnaire to calculate bleeding score (sum of daily ratings of blood loss) and health-related QoL<br>[61]				• The numbers of women returning questionnaires (69.1%) failed to meet power requirements
MBL score (based on number and absorbency of products used and self-perceived "heavy" days)<br>[62]	• Ferritin<br>• Platelet count	• Validation of discriminatory power<br>• Internal consistency<br>• Validated for pads (mini, normal, super, night/superplus) and tampons (mini, regular, super, superplus); brand not specified	• Easy, practical, simple, feasible<br>• Highly reliable<br>• Does not rely on participant's memory	• Generalizability unknown
Menorrhagia-specific QoL questionnaire, PBAC, and SF-36 (Iranian version)<br>[63]		• External validation (Iranian population)<br>• Validated for medium-sized Panberes sanitary towels		• Limitations in assessing some problems that patients with HMB may experience
Internet-based survey (about predefined symptoms of HMB, consultation with physician, and effect on daily life)<br>[64]		• Validated for unspecified pads and tampons	• Internet-based and paper surveys conducted when people do not have the same access to information technology give similar outcomes; thus, it is unlikely that the internet-based medium affected the results	• Information was only from the patient's perspective not from that of the healthcare professional
MBQ (scores on heaviness, pain, irregularity, and QoL)	• SF-36	• Validation of discriminatory power<br>• Internal consistency<br>• Content validation	• Covers a range of symptoms, the amount and regularity of bleeding, and the impact of symptoms on women's lives, specifically on the fear of social embarrassment based on focus group studies	• Generalizability unknown

Study (reference)	Concurrent validation (reference)	Other validation	Advantages	Disadvantages
[65]		• Validation of 1-month recall	• A systematic process was used to develop the questionnaire using real input from women with HMB across a range of patient experiences<br>• Accurately summarizes the experiences of participants prospectively catalogued over 1 month<br>• Could improve the evaluation of women with self-reported HMB in clinical practice<br>• Could facilitate interpretation of data on treatment effectiveness and facilitate comparison and summation of results across studies	
Female Health Questionnaire, online and paper versions – based on Fraser et al., 2015 (36)<br>[66]			• Mixed mode (online and paper) strategy used to limit bias	• Subject to recall bias<br>• Online survey more likely to be completed by females with menstrual cycle issues<br>• Presence of illness or the use of medication not recorded; these could increase HMB diagnosis
Additional methods involving self-perception				
Subjective complaint of HMB<br>[67]	• AH method [1]	• Validation of discriminatory power		
Subjective assessment of MBL<br>[68]	• AH method [1]	• Validation of discriminatory power<br>• Validated for unspecified pads and tampons	• Quick	• Does not take clots into account<br>• Many women are not good judges of the volume of their MBL
Subjective complaint and clinical history of HMB, and subjective daily assessment of MBL on a 4-point scale<br>[69]	• AH method [3]<br>• Duration of menstruation	• Validation of discriminatory power		• May result in large numbers of hysterectomies for HMB that cannot be objectively confirmed<br>• Not as accurate as AH method
Subjective and objective clinical parameters<br>[70]	• AH method [1]	• Validation of discriminatory power<br>• Validated for unspecified pads and tampons	• Hemoglobin levels fall with increasing blood loss, although the relationship is insufficiently precise for this to be a diagnostic test for objective HMB	• It is difficult to establish the volume of MBL with certainty without an objective assessment<br>• There was substantial variation in the number of sanitary items used<br>• For the hemoglobin test, HMB

Study (reference)	Concurrent validation (reference)	Other validation	Advantages	Disadvantages
				can coexist with a hemoglobin level well within the normal range, so it should not be relied on as a screening test to deny treatment<br>• The relationship between objective MBL and height is weak and of doubtful clinical importance
Mixed linear model (menstrual diary and laboratory parameters) [71]	• AH method [3]	• Validation of discriminatory power<br>• External validation (similar study population)	• Simple and accurate<br>• A menstruation diary could be a better descriptor of blood loss measurements than the AH method<br>• The specificity and sensitivity make this technique suitable for clinical use and counselling<br>• Can be used to estimate MBL in clinical studies	• Does not give a precise MBL measure; in studies where MBL is the primary efficacy outcome, the AH method will remain the gold standard (although it does not account for MBL outside sanitary protection)<br>• Further studies are required to test reproducibility and generalizability

AH = alkaline hematin; BQ = bleeding questionnaire; HMB = heavy menstrual bleeding; MBL = menstrual blood loss; MBQ = Menstrual Bleeding Questionnaire; MEQ = Menstrual Evaluation Questionnaire; MFL = menstrual fluid loss; MIQ = Menorrhagia Impact Questionnaire; MVJ = Mansfield–Voda–Jorgensen Menstrual Bleeding Scale; PBAC = pictorial blood loss assessment chart; QoL = quality of life; SAP-c = superabsorbent polymer-containing; SF-36 = Medical Outcomes Study Short 36-item Short Form Health Survey.

References

1. Hallberg L, Nilsson L. Determination of menstrual blood loss. Scand J Clin Lab Invest. 1964;16:244–248.
2. Shaw ST, Jr., Aaronson DE, Moyer DL. Quantitation of menstrual blood loss–further evaluation of the alkaline hematin method. Contraception. 1972;5:497–513.
3. Newton J, Barnard G, Collins W. A rapid method for measuring menstrual blood loss using automatic extraction. Contraception. 1977;16:269–282.
4. Desai H, Raghavan KS, Mapa MK, Gupta AN. Quantitative estimation of menstrual blood loss. Ind J Med Res. 1982;75:827–830.
5. van Eijkeren MA, Scholten PC, Christiaens GC, Alsbach GP, Haspels AA. The alkaline hematin method for measuring menstrual blood loss–a modification and its clinical use in menorrhagia. Eur J Obstet Gynecol Reprod Biol. 1986;22:345–51.
6. Vasilenko P, Kraicer PF, Kaplan R, deMasi A, Freed N. A new and simple method of measuring menstrual blood loss. J Reprod Med Obstet Gynecol. 1988;33:293–297.
7. Gannon MJ, Day P, Hammadieh N, Johnson N. A new method for measuring menstrual blood loss and its use in screening women before endometrial ablation. Br J Obstet Gynaecol. 1996;103:1029–1033.
8. Hurskainen R, Teperi J, Turpeinen U, Grenman S, Kivela A, Kujansuu E, et al. Combined laboratory and diary method for objective assessment of menstrual blood loss. Acta Obstet Gynecol Scand. 1998;77:201–204.
9. Magnay JL, Nevatte TM, Dhingra V, O'Brien S. Menstrual blood loss measurement: validation of the alkaline hematin technique for feminine hygiene products containing superabsorbent polymers. Fertil Steril. 2010;94:2742–2746.
10. Magnay JL, Schonicke G, Nevatte TM, O'Brien S, Junge W. Validation of a rapid alkaline hematin technique to measure menstrual blood loss on feminine towels containing superabsorbent polymers. Fertil Steril. 2011;96:394–398.
11. Reid PC, Virtanen-Kari S. Randomised comparative trial of the levonorgestrel intrauterine system and mefenamic acid for the treatment of idiopathic menorrhagia: a multiple analysis using total menstrual fluid loss, menstrual blood loss and pictorial blood loss assessment charts. Br J Obstet Gynaecol. 2005;112:1121–5.
12. Fraser IS, Warner P, Marantos PA. Estimating menstrual blood loss in women with normal and excessive menstrual fluid volume. Obstet Gynecol. 2001;98:806–814.
13. Gudmundsdottir BR, Hjaltalin EF, Bragadottir G, Hauksson A, Geirsson RT, Onundarson PT. Quantification of menstrual flow by weighing protective pads in women with normal, decreased or increased menstruation. Acta Obstet Gynecol Scand. 2009;88:275–279.
14. Gleeson N, Devitt M, Buggy F, Bonnar J. Menstrual blood loss measurement with Gynaeseal. Aus New Zeal J Obstet Gynaecol. 1993;33:79–80.
15. Stewart K, Greer R, Powell M. Women's experience of using the Mooncup. J Obstet Gynaecol. 2010;30:285–287.
16. Baldwin RM, Whalley PJ, Pritchard JA. Measurements of menstrual blood loss. Am J Obstet Gynecol. 1961;81:739–742.
17. Tauxe WN. Quantitation of menstrual blood loss: a radioactive method utilizing a counting dome. J Nucl Med. 1962;3:282–287.
18. Price DC, Forsyth EM, Cohn SH, Cronkite EP. The study of menstrual and other blood loss, and consequent iron deficiency, by Fe^{59} whole-body counting. Can Med Assoc J. 1964;90:51–4.
19. Cheyne GA, Shepherd MM. Comparison of chemical and atomic absorption methods for estimating menstrual blood loss. J Med Lab Technol. 1970;27:350–354.

20. Napolitano M, Dolce A, Celenza G, Grandone E, Perilli MG, Siragusa S, et al. Iron-dependent erythropoiesis in women with excessive menstrual blood losses and women with normal menses. Ann Hematol. 2014;93:557–63.
21. Higham JM, O'Brien PMS, Shaw RW. Assessment of menstrual blood loss using a pictorial chart. Br J Obstet Gynaecol. 1990;97:734–739.
22. Deeny M, Davis JA. Assessment of menstrual blood loss in women referred for endometrial ablation. Eur J Obstet Gynecol Reprod Biol. 1994;57:179–180.
23. Janssen CAH, Scholten PC, Heintz APM. A simple visual assessment technique to discriminate between menorrhagia and normal menstrual blood loss. Obstet Gynecol. 1995;85:977–982.
24. Shapley M, Redman CWE. Assessment of menstrual blood loss using a pictorial chart and endometrial sampling within the community. J Obstet Gynaecol. 1995;15:123–124.
25. Barrington JW, Bowen-Simpkins P. The levonorgestrel intrauterine system in the management of menorrhagia. Br J Obstet Gynaecol. 1997;104:614–16.
26. Barr F, Brabin L, Agbaje O. A pictorial chart for managing common menstrual disorders in Nigerian adolescents. Int J Gynecol Obstet. 1999;66:51–53.
27. Reid PC, Coker A, Coltart R. Assessment of menstrual blood loss using a pictorial chart: a validation study. Br J Obstet Gynaecol. 2000;107:320–322.
28. Biri A, Bozkurt N, Korucuoglu U, Yilmaz E, Tiras B, Guner H. Use of pictorial chart for managing menorrhagia among Turkish women. J Turk Ger Gynecol Assoc. 2007;9:35–37.
29. Zakherah MS, Sayed GH, El-Nashar SA, Shaaban MM. Pictorial blood loss assessment chart in the evaluation of heavy menstrual bleeding: diagnostic accuracy compared to alkaline hematin. Gynecol Obstet Invest. 2011;71:281–284.
30. Nahidi F, Bagheri L, Jannesari S, Majd HA. Relationship between delivery type and menstrual disorders: a case–control study. J Res Health Sci. 2011;11:83–90.
31. Sanchez J, Andrabi S, Bercaw JL, Dietrich JE. Quantifying the PBAC in a pediatric and adolescent gynecology population. Pediatr Hematol Oncol. 2012;29:479–484.
32. Hald K, Lieng M. Assessment of periodic blood loss: interindividual and intraindividual variations of pictorial blood loss assessment chart registrations. J Min Invas Gynecol. 2014;21:662–668.
33. MacEachern K, Kaur H, Toukh M, Mumal I, Hamilton A, Scovil S, et al. Comprehensive evaluation of hemostasis in normal women: impact on the diagnosis of mild bleeding disorders. Clin Appl Thromb Hemost. 2015;21:72–81.
34. Wyatt KM, Dimmock PW, Walker TJ, O'Brien PMS. Determination of total menstrual blood loss. Fertil Steril. 2001;76:125–131.
35. Wyatt KM, Dimmock PW, Hayes-Gill B, Crowe J, O'Brien PMS. Menstrual symptometrics: A simple computer-aided method to quantify menstrual cycle disorders. Fertil Steril. 2002;78:96–101.
36. Larsen L, Coyne K, Chwalisz K. Validation of the menstrual pictogram in women with leiomyomata associated with heavy menstrual bleeding. Reprod Sci. 2013;20:680–687.
37. Magnay JL, Nevatte TM, Seitz C, O'Brien S. A new menstrual pictogram for use with feminine products that contain superabsorbent polymers. Fertil Steril. 2013;100:1715–21.e1–4.
38. Magnay JL, Nevatte TM, O'Brien S, Gerlinger C, Seitz C. Validation of a new menstrual pictogram (superabsorbent polymer-c version) for use with ultraslim towels that contain superabsorbent polymers. Fertil Steril. 2014;101:515–522.e5.
39. Sowter M, Bidgood K, Richardson J. A comparative randomised trial of the effect of preoperative endometrial inhibition on the long-term outcome of transcervical endometrial resection. Gynaecol Endosc. 1997;6:33–37.
40. Shaw RW, Brickley MR, Evans L, Edwards MJ. Perceptions of women on the impact of menorrhagia on their health using multi-attribute utility assessment. Br J Obstet Gynaecol. 1998;105:1155–9.

41. Heath ALM, Skeaff CM, Gibson RS. Validation of a questionnaire method for estimating extent of menstrual blood loss in young adult women. J Trace Elem Med Biol. 1999;12:231–235.
42. Hodgson DA, Feldberg IB, Sharp N, Cronin N, Evans M, Hirschowitz L. Microwave endometrial ablation: development, clinical trials and outcomes at three years. Br J Obstet Gynaecol. 1999;106:684–94.
43. Johannes CB, Crawford SL, Woods J, Goldstein RB, Tran D, Mehrotra S, et al. An electronic menstrual cycle calendar: comparison of data quality with a paper version. Menopause. 2000;7:200–8.
44. Harlow B, Missmer S, Cramer D, Barbieri R. Does tubal sterilization influence the subsequent risk of menorrhagia or dysmenorrhea? Fertil Steril. 2002;77:754–760.
45. Côté I, Jacobs P, Cumming D. Use of health services associated with increased menstrual loss in the United States. Am J Obstet Gynecol. 2003;199:343–348.
46. Shapley M, Jordan K, Croft PR. Increased vaginal bleeding and psychological distress: a longitudinal study of their relationship in the community. BJOG. 2003;110:548–54.
47. Mansfield PK, Voda A, Allison G. Validating a pencil-and-paper measure of perimenopausal menstrual blood loss. Women's Health Issues. 2004;14:242–247.
48. Warner PE, Critchley HOD, Lumsden MA, Campbell-Brown M, Douglas A, Murray GD. Menorrhagia I: measured blood loss, clinical features, and outcome in women with heavy periods – a survey with follow-up data. Am J Obstet Gynecol. 2004;190:1216–1223.
49. Santer M, Warner P, Wyke S. A Scottish postal survey suggested that the prevailing clinical preoccupation with heavy periods does not reflect the epidemiology of reported symptoms and problems. J Clin Epidemiol. 2005;58:1206–10.
50. Lee LK, Chen PCY, Lee KK, Kaur J. Menstruation among adolescent girls in Malaysia: a cross-sectional school survey. Singapore Med J. 2006;47:869–874.
51. Pisoni CN, Cuadrado MJ, Khamashta MA, Hunt BJ. Treatment of menorrhagia associated with oral anticoagulation: efficacy and safety of the levonorgestrel releasing intrauterine device (Mirena coil). Lupus. 2006;15:877–80.
52. Santer M, Wyke S, Warner P. What aspects of periods are most bothersome for women reporting heavy menstrual bleeding? Community survey and qualitative study. BMC Women's Health. 2007;7:8.
53. Pawar A, Krishnan R, Davis K, Bosma K, Kulkarni R. Perceptions about quality of life in a school-based population of adolescents with menorrhagia: implications for adolescents with bleeding disorders. Haemophilia. 2008;14:579–83.
54. Lukes AS, Muse K, Richter HE, Moore KA, Patrick DL. Estimating a meaningful reduction in menstrual blood loss for women with heavy menstrual bleeding. Curr Med Res Opin. 2010;26:2673–2678.
55. Bushnell DM, Martin ML, Moore KA, Richter HE, Rubin A, Patrick DL. Menorrhagia Impact Questionnaire: assessing the influence of heavy menstrual bleeding on quality of life. Curr Med Res Opin. 2010;26:2745–2755.
56. de Souza SS, Camargos AF, Ferreira MC, de Assis Nunes Pereira F, de Rezende CP, Araujo CA, et al. Hemoglobin levels predict quality of life in women with heavy menstrual bleeding. Arch Gynecol Obstet. 2010;281:895–900.
57. Deal LS, Williams VS, Fehnel SE. Development of an electronic daily uterine fibroid symptom diary. Patient. 2011;4:31–44.
58. Revel-Vilk S, Paltiel O, Lipschuetz M, Ilan U, Hyam E, Shai E, et al. Underdiagnosed menorrhagia in adolescents is associated with underdiagnosed anemia. J Pediatr. 2012;160:468–472.
59. Rae C, Furlong W, Horsman J, Pullenayegum E, Demers C, St-Louis J, et al. Bleeding disorders, menorrhagia and iron deficiency: impacts on health-related quality of life. Haemophilia. 2013;19:385–91.

60. Karlsson TS, Marions LB, Edlund MG. Heavy menstrual bleeding significantly affects quality of life. Acta Obstet Gynecol Scand. 2014;93:52–7.
61. Sambrook AM, Elders A, Cooper KG. Microwave endometrial ablation versus thermal balloon endometrial ablation (MEATBall): 5-year follow up of a randomised controlled trial. BJOG. 2014;121:747–53.
62. Toxqui L, Perez-Granados AM, Blanco-Rojo R, Wright I, Vaquero MP. A simple and feasible questionnaire to estimate menstrual blood loss: relationship with hematological and gynecological parameters in young women. BMC Women's Health. 2014;14 (1):71.
63. Goshtasebi A, Mazari Z, Behboudi Gandevani S, Naseri M. Anti-hemorrhagic activity of Punica granatum L. flower (Persian Golnar) against heavy menstrual bleeding of endometrial origin: a double-blind, randomized controlled trial. Med J Islam Repub Iran. 2015;29:199.
64. Fraser IS, Mansour D, Breymann C, Hoffman C, Mezzacasa A, Petraglia F. Prevalence of heavy menstrual bleeding and experiences of affected women in a European patient survey. Int J Gynaecol Obstet. 2015;128:196–200.
65. Matteson KA, Scott DM, Raker CA, Clark MA. The menstrual bleeding questionnaire: development and validation of a comprehensive patient-reported outcome instrument for heavy menstrual bleeding. Br J Obstet Gynaecol. 2015;122:681–9.
66. Bruinvels G, Burden R, Brown N, Richards T, Pedlar C. The prevalence and impact of heavy menstrual bleeding (menorrhagia) in elite and non-elite athletes. PLoS One. 2016;11:e0149881.
67. Haynes PJ, Hodgson H, Anderson AB, Turnbull AC. Measurement of menstrual blood loss in patients complaining of menorrhagia. Br J Obstet Gynaecol. 1977;84:763–8.
68. Chimbira TH, Anderson ABM, Turnbull AC. Relation between measured menstrual blood loss and patient's subjective assessment of loss, duration of bleeding, number of sanitary towels used, uterine weight and endometrial surface area. Br J Obstet Gynaecol. 1980;87:603–9.
69. Fraser IS, McCarron G, Markham R. A preliminary study of factors influencing perception of menstrual blood loss volume. Am J Obstet Gynecol. 1984;149:788–93.
70. Higham JM, Shaw RW. Clinical associations with objective menstrual blood volume. Eur J Obstet Gynecol Reprod Biol. 1999;82:73–76.
71. Schumacher U, Schumacher J, Mellinger U, Gerlinger C, Wienke A, Endrikat J. Estimation of menstrual blood loss volume based on menstrual diary and laboratory data. BMC Women's Health. 2012;12 24.
