# Supplementary material for: A systematic review of methods to measure menstrual blood loss
Source: BMC Womens Health. 2018 Aug 22;18:142. doi: 10.1186/s12905-018-0627-8 (PMC6106944; doi:10.1186/s12905-018-0627-8)
Supplement: Supplementary file 4 — Table S4. Further validation of methods. (PDF 171 kb) [file 12905_2018_627_MOESM4_ESM.pdf]

A systematic review of methods to measure menstrual blood loss

SUPPLEMENTAL TABLE 4

Further validation of methods.

Author and year (reference)	Method	Study population	N (n)[a]	Recovery of blood/iron	Internal consistency	Other validation
**AH**						
Magnay et al., 2011 [1]	Modified AH method (rapid AH technique for SAP-c products)	Simulated menstrual fluid	N/A (63 towels with simulated fluid samples; 21 for each type)	Linearity was confirmed, and ≥85% blood recovery was reproducibly achieved from 0.05–30 mL at 5–100% simulated menstrual fluid compositions (except at low volume/high dilution equivalent to <4 mL)<br><br>Mean extraction efficiency (with absorbency conversion factors): normal, 91.3%; long, 94.3%; night, 94.5%<br><br>There was no significant difference in hemoglobin recovery between towel types (one-way ANOVA, P>.05)	Intra-assay coefficient of variation was <4.5% for both 5 and 20 mL volumes of blood applied to normal towels (n = 20)	Linear regression analysis of reference versus test method gave a coefficient of determination (r^2) of 0.991 (P<.0001, n = 63)
Magnay et al. 2010 [2]	Modified AH (SAP version)	Simulated menstrual fluid	N/A (6/10 simulated fluid samples for each experiment)	90% recovery was reproducibly achieved for up to 30 mL of applied volume (n = 10/11 per volume) and at all tested simulated menstrual fluid compositions (n = 6 per composition), except at low volume or high dilution equivalent to <2 mL	Precision improved as the applied volume increased from 5 mL to 20/30 mL (10 replicates)<br><br>Coefficients of variation (10 replicates): normal, 10.6–6.3; long, 13.4–5.6; night, 9.4–6.0	–
Hurskainen et al., 1998 [3]	Modified AH method	N/A	N/A (blood)	Mean blood recovery of 10–20 mL from tampons and 10–	–	–

Author and year (reference)	Method	Study population	N (n)[a]	Recovery of blood/iron	Internal consistency	Other validation
	(Stomacher® blender and absorbance at A_{564}; menstrual diary)			200 mL from towels (n = 3 per volume): 93%		
Gannon et al., 1996 [4]	Modified AH method (detergent extraction; photometric analysis; calibration factor)	Women with heavy MBL	146 (292) (time-expired whole blood)	There was a linear relationship between 10 and 250 mL of blood (gradient, 1.0; n = 3 per volume)	There was good intra-individual correlation between two repeat measurements (r = 0.713, P<.001)	–
Vasilenko et al., 1988 [5]	Modified AH method (volumetric test; non-caustic chemicals; standard curves)	Women with normal MBL and those diagnosed with dysfunctional uterine bleeding	10 (35) (whole blood solution; hematocrit 50%)	There was a significant linear relationship between 0 and 10 mL of blood (P<.0001) for the four products; on average, extraction efficiency was 96.9%<br><br>Thin maxi pads had a significantly lower slope than blood alone (P<.001); extraction efficiency: 7.5 mL, 85.2%; 10.0 mL, 74.8%<br><br>Extraction efficiencies of both brands of tampons were 99.0%	The variation in individuals from menstrual period to period (1.5 SD units) was as great as the variation between women (1.5 SD units); thus, it is advisable to determine blood losses from more than one cycle per woman	–
van Eijkeren et al., 1986 [6]	Modified AH method (Stomacher blender and modified equation for calculation of MBL)	Women with self-perceived heavy MBL	21 (21) (outdated whole blood)	Mean recovery of blood from tampons and towels (using equation that subtracts blanks and incorporates post-filtration volume; n = 3–4 per volume):<br>10 mL, 98%<br>40 mL, 102%<br>80 mL, 98%<br>140 mL, 99%<br>200 mL, 98%	The coefficient of variation using this technique varied between 6.1% and 7.5%<br><br>The intra-assay variability (variation on one day) was 3.3%; the inter-assay variability (variation over several days) was 3.8%<br><br>Variation between observers was negligible	–

Author and year (reference)	Method	Study population	N (n)[a]	Recovery of blood/iron	Internal consistency	Other validation
Desai et al., 1982 [7]	Modified AH method (Stomacher blender; automation)	N/A	N/A (blood)	Both types of pads, "gave close to 100% recovery", using 500 mL of sodium hydroxide and 15 min of extraction per pad	Intra-individual and intracycle variation was low	External validation (Indian population)
Newton et al., 1977 [8]	Modified AH method (Stomacher blender; automation)	N/A	N/A (peripheral blood)	Mean recovery of blood (5–200 mL; n = 4 per volume) from seven tampons and pads was 82–93% (coefficient of variation on replicate analysis was 3.4–11.0%)<br><br>Mean recovery of 20 mL of blood added to each of 13 products (n = 4): 20.3 mL (range, 18.4–22.3 mL)	–	–
Shaw et al., 1972 [9]	Modified AH method (increased incubation time)	N/A	N/A (outdated whole blood)	Recovery of blood (n = 3):<br>Pads:<br>0.1 mL, 95–103%<br>0.5 mL, 98–101%<br>Pads and tampons:<br>1.0 mL, 95–100%<br>2.0 mL, 98–100%<br>3.0 mL, 100–104%<br>4.0 mL, 96–105%<br>5.0 mL, 96–100%<br><br>Average recovery of vaginal MBL from tampons after insertion for 6–16 h: 102.5% (range, 100–107%; n = 6)	–	–
Hallberg & Nilsson, 1964 [10]	AH	Healthy women tested the accuracy of the method	2 (24) (simulated fluid samples)	Recovery of dried blood (n = 10 per test):<br>After 5 h, 87.4% (SE, 1.0)<br>After 20 h, 96.3% (SE, 0.5)	Consistent losses were seen over 12 cycles in two women	–
**MFL**						
Gudmundsdottir et al., 2009 [11]	MFL	Women with diagnosed heavy MBL or normally menstruating	78 (78)	–	–	External validation (adults vs teenagers; users of oral contraceptives vs non-users)

Author and year (reference)	Method	Study population	N (n)[a]	Recovery of blood/iron	Internal consistency	Other validation
		women/adolescents				
Fraser et al., 2001 [12]	Regression estimation of MBL from total MFL	Women with self-perceived normal MBL, heavy MBL, or previous heavy MBL	24 (48)	–	Median inter-cycle variability:<br>MBL, 18%<br>MFL, 26%	–
**Measurement of iron/labelled blood**						
Cheyne & Shepherd, 1970 [13]	Chemical [14] and atomic absorption spectrophoto-metry analysis of iron recovery	Menstruating women	N/A<br>(25 used pads)	Recovery of added iron (n = 3–4):<br>atomic absorption, 97.5–105%<br>chemical determination, 98.1–100.9%	SD of difference in duplicate estimations among six washes:<br>either method,<br>0.1 mg Fe/100 mL<br>Average SD from mean, ±3%<br><br>SD of differences in samples (mg Fe/100 mL):<br>atomic absorption, 0.029<br>chemical, 0.067<br>Average SD from mean (%):<br>atomic absorption, ±0.90<br>chemical, ±2.24	–
Tauxe, 1962 [15]	Radioactivity counting dome	N/A	N/A	–	Experimental accuracy of counting dome from series of six different quantities of venous blood, 2 SDs = 1.72 (1.6% of mean 104.89 mL)<br><br>0.8% error of radioactivity over 10 different sites on the dome	–
Baldwin et al., 1961 [16]	Fe^{59} radioactive iron	N/A	N/A<br>(pooled blood)	–	The error in recovery of blood samples was consistently less than 0.5 mL per 20 mL	–
**PBAC**						
MacEachern et al., 2015 [17]	e-PBAC<br>e-BQ	Women without menstrual disorder	47 (47)	–	–	External validation (oral contraceptive users vs non-users)
Goshtasebi et al., 2015 [18]	PBAC<br>SF-36	Women with self-perceived HMB	76 (304)	–		External validation (Iranian women)
Hald & Lieng, 2014 [19]	Modified PBAC (revised icons)	Untreated women with self-perceived light, normal, or	118 (236)	–	Intra-class correlation coefficient for PBAC	–

Author and year (reference)	Method	Study population	N (n)[a]	Recovery of blood/iron	Internal consistency	Other validation
		heavy MBL reporting on two cycles			measurements in two cycles, 0.86<br>Inter-individual variation in PBAC scores was high	
Revel-Vilk et al., 2012 [20]	PBAC and HSC bleeding questionnaire	Adolescent girls	66 (66)	–	–	External validation (adolescents)
Sanchez et al., 2012 [21]	PBAC	Menstruating adolescents	73 (73)	–	–	External validation (adolescents)
Nahidi et al., 2011 [22]	Modified PBAC (revised icons, volume loss)	Women with or without menstrual disorder	160 (320)	–	–	External validation (Iranian women)
Biri et al., 2007 [23]	PBAC [24]	Menstruating women	600 (600)	–	–	External validation (PBAC in Turkish women)
Shapley & Redman, 1995 [25]	PBAC	Women aged ≥40 years	283 (283)	–	–	PBAC may overestimate MBL in the general community
Janssen et al., 1995 [26]	Modified PBAC	Women with self-perceived normal or heavy MBL or with unexplained anemia	288 (489)	–	85% of participants were consistent in assessment of their second menses	Agreement between participant and investigator scores was good
Higham et al., 1990 [27]	PBAC	Women with a range of MBLs	28 (55)	–	–	Agreement between gynecologist and participant scores was good (within 2 SDs)
**Menstrual pictogram**						
Magnay et al., 2014 [28]	Menstrual pictogram (SAP version)	Women with self-perceived light, normal, or heavy MBL	119 (235)	–	–	Participant and expert ratings concurred for 59% (1,957/3,315) of towels (κ = 0.46); in 95% of cases the scoring discrepancy was less than or equal to one icon category
Larsen et al., 2013 [29]	Modified menstrual pictogram (excluding extraneous MBL)	Women with confirmed HMB	170 (169)	–	–	External validation (North American treated population)
Magnay et al., 2013	Menstrual	Premenopausal women with	12	There was a significant	There was a high level of	There was no significant

Author and year (reference)	Method	Study population	N (n)[a]	Recovery of blood/iron	Internal consistency	Other validation
[30]	pictogram (SAP version)	regular menstrual cycles who had not previously used a graphical method to assess menstrual loss	(N/A) (simulated menstrual fluid)	correlation between the volume of blood applied and that recovered by AH (r^2 = 0.992, *P*<.0001), and between the volume of fluid applied and the soiled towel weight (r^2 = 0.998, *P*<.0001)	agreement between individual scores: for 112 (83%) of 135 towels, the participants' readings either completely concurred or differed by only a single icon; there was no significant difference between the 12 sets of scores (ANOVA on ranks, *P*>.05)	difference between expert and participant ratings (ANOVA on ranks, *P*>.05; n = 135 towels)
Wyatt et al., 2002 [31]	Menstrual pictogram Symptometrics device	Women with self-perceived normal or heavy MBL	59 (109)	–	–	Overall agreement between paper assessment charts for MBL: cycle 1, 92%; cycle 2, 92%; κ: cycle 1, 0.84 (*P*<.00001); cycle 2, 0.81 (*P*<.00001)
**Methods involving self-perception of MBL**						
Matteson et al., 2015 [32]	MBQ	Non-pregnant women with and without self-reported HMB	27 (–)	–	MBQ domains were internally consistent (Cronbach's alpha = 0.87–0.94)	–
			13 (–)	–	There was good-to-excellent correlation of daily, weekly, and monthly recall scores (ρ = 0.80–0.92)	–
Toxqui et al., 2014 [33]	MBL score	Healthy women	165 (–)	–	Cronbach's alpha coefficient for MBL score was 0.83	–
Rae et al., 2013 [34]	Health Utilities Index questionnaire	Women with von Willebrand disease	185 (185)	–	–	External validation (von Willebrand disease)
Bushnell et al., 2010 [35]	MIQ	Women with self-perceived normal MBL or diagnosed heavy MBL	262 (262)	–	With one exception, all intra-class correlation coefficients (reflecting test–retest reliability) for treatments and controls were >0.70 (n = 131)	Correlations across MIQ and to other questionnaires were almost all highly significant (*P*<.001)
de Souza et al. 2010 [36]	SF-36	Women with heavy MBL defined as >80 mL for ≥3 months	58 (58)	–	–	External validation (Portuguese women)
Lee et al., 2006 [37]	Question-naire (MBL, number of pads)	Adolescent girls	2411 (–)	–	–	External validation (Malaysian adolescents)

Author and year (reference)	Method	Study population	N (n)[a]	Recovery of blood/iron	Internal consistency	Other validation
Pawar et al., 2008 [38]	QoL questionnaire	Adolescent women	45 (–)	–	–	External validation (US adolescents)
Warner et al., 2004 [39]	MEQ	Women with self-perceived menstrual complaint	209 (N/A)	–	Compared with the previous 6 months the collected period was reported as: more in amount, 5%; the same, 16%; a bit less, 42%; much less, 36%	–
Johannes et al., 2000 [40]	A prototype electronic menstrual calendar and an identical paper calendar	Women with self-perceived normal MBL	24 (N/A)	–	–	Overall, participants preferred the electronic version (69.6% vs 21.7%; *P*=.006)
Haynes et al. 1977 [41]	Subjective complaint	Women with self-perceived heavy MBL (25) and women with normal MBL (12)	37 (>269)	–	–	Compared to women with normal MBL there was no difference in the coefficient of variation for MBL from one period to the next

[a]N = study population size; n = number of cycles studied.

AH = alkaline hematin; ANOVA = analysis of variance; BQ = bleeding questionnaire; HMB = heavy menstrual bleeding; HSC = Hospital for Sick Children; MBL = menstrual blood loss; MBQ = Menstrual Bleeding Questionnaire; MEQ = Menstrual Evaluation Questionnaire; MFL = menstrual fluid loss; MIQ = Menorrhagia Impact Questionnaire; PBAC = pictorial blood loss assessment chart; SAP = superabsorbent polymer; SD = standard deviation; SE = standard error; SF-36 = Medical Outcomes Study 36-item Short Form Health Survey.

References

1. Magnay JL, Schonicke G, Nevatte TM, O'Brien S, Junge W. Validation of a rapid alkaline hematin technique to measure menstrual blood loss on feminine towels containing superabsorbent polymers. Fertil Steril. 2011;96:394–398.
2. Magnay JL, Nevatte TM, Dhingra V, O'Brien S. Menstrual blood loss measurement: validation of the alkaline hematin technique for feminine hygiene products containing superabsorbent polymers. Fertil Steril. 2010;94:2742–2746.
3. Hurskainen R, Teperi J, Turpeinen U, Grenman S, Kivela A, Kujansuu E, et al. Combined laboratory and diary method for objective assessment of menstrual blood loss. Acta Obstet Gynecol Scand. 1998;77:201–204.
4. Gannon MJ, Day P, Hammadieh N, Johnson N. A new method for measuring menstrual blood loss and its use in screening women before endometrial ablation. Br J Obstet Gynaecol. 1996;103:1029–1033.
5. Vasilenko P, Kraicer PF, Kaplan R, deMasi A, Freed N. A new and simple method of measuring menstrual blood loss. J Reprod Med Obstet Gynecol. 1988;33:293–297.
6. van Eijkeren MA, Scholten PC, Christiaens GC, Alsbach GP, Haspels AA. The alkaline hematin method for measuring menstrual blood loss–a modification and its clinical use in menorrhagia. Eur J Obstet Gynecol Reprod Biol. 1986;22:345–51.
7. Desai H, Raghavan KS, Mapa MK, Gupta AN. Quantitative estimation of menstrual blood loss. Ind J Med Res. 1982;75:827–830.
8. Newton J, Barnard G, Collins W. A rapid method for measuring menstrual blood loss using automatic extraction. Contraception. 1977;16:269–282.
9. Shaw ST, Jr., Aaronson DE, Moyer DL. Quantitation of menstrual blood loss–further evaluation of the alkaline hematin method. Contraception. 1972;5:497–513.
10. Hallberg L, Nilsson L. Determination of menstrual blood loss. Scand J Clin Lab Invest. 1964;16:244–248.
11. Gudmundsdottir BR, Hjaltalin EF, Bragadottir G, Hauksson A, Geirsson RT, Onundarson PT. Quantification of menstrual flow by weighing protective pads in women with normal, decreased or increased menstruation. Acta Obstet Gynecol Scand. 2009;88:275–279.
12. Fraser IS, Warner P, Marantos PA. Estimating menstrual blood loss in women with normal and excessive menstrual fluid volume. Obstet Gynecol. 2001;98:806–814.
13. Cheyne GA, Shepherd MM. Comparison of chemical and atomic absorption methods for estimating menstrual blood loss. J Med Lab Technol. 1970;27:350–354.
14. Ramsay WN. Iron metabolism and haemoglobin formation in the embryonated hen egg. 2. Some observations on the embryonic blood supply. Biochem J. 1951;49:494–9.
15. Tauxe WN. Quantitation of menstrual blood loss: a radioactive method utilizing a counting dome. J Nucl Med. 1962;3:282–287.
16. Baldwin RM, Whalley PJ, Pritchard JA. Measurements of menstrual blood loss. Am J Obstet Gynecol. 1961;81:739–742.
17. MacEachern K, Kaur H, Toukh M, Mumal I, Hamilton A, Scovil S, et al. Comprehensive evaluation of hemostasis in normal women: impact on the diagnosis of mild bleeding disorders. Clin Appl Thromb Hemost. 2015;21:72–81.
18. Goshtasebi A, Mazari Z, Behboudi Gandevani S, Naseri M. Anti-hemorrhagic activity of Punica granatum L. flower (Persian Golnar) against heavy menstrual bleeding of endometrial origin: a double-blind, randomized controlled trial. Med J Islam Repub Iran. 2015;29:199.
19. Hald K, Lieng M. Assessment of periodic blood loss: interindividual and intraindividual variations of pictorial blood loss assessment chart registrations. J Min Invas Gynecol. 2014;21:662–668.

20. Revel-Vilk S, Paltiel O, Lipschuetz M, Ilan U, Hyam E, Shai E, et al. Underdiagnosed menorrhagia in adolescents is associated with underdiagnosed anemia. J Pediatr. 2012;160:468–472.
21. Sanchez J, Andrabi S, Bercaw JL, Dietrich JE. Quantifying the PBAC in a pediatric and adolescent gynecology population. Pediatr Hematol Oncol. 2012;29:479–484.
22. Nahidi F, Bagheri L, Jannesari S, Majd HA. Relationship between delivery type and menstrual disorders: a case–control study. J Res Health Sci. 2011;11:83–90.
23. Biri A, Bozkurt N, Korucuoglu U, Yilmaz E, Tiras B, Guner H. Use of pictorial chart for managing menorrhagia among Turkish women. J Turk Ger Gynecol Assoc. 2007;9:35–37.
24. Higham JM, Shaw RW. Clinical associations with objective menstrual blood volume. Eur J Obstet Gynecol Reprod Biol. 1999;82:73–76.
25. Shapley M, Redman CWE. Assessment of menstrual blood loss using a pictorial chart and endometrial sampling within the community. J Obstet Gynaecol. 1995;15:123–124.
26. Janssen CAH, Scholten PC, Heintz APM. A simple visual assessment technique to discriminate between menorrhagia and normal menstrual blood loss. Obstet Gynecol. 1995;85:977–982.
27. Higham JM, O'Brien PMS, Shaw RW. Assessment of menstrual blood loss using a pictorial chart. Br J Obstet Gynaecol. 1990;97:734–739.
28. Magnay JL, Nevatte TM, O'Brien S, Gerlinger C, Seitz C. Validation of a new menstrual pictogram (superabsorbent polymer-c version) for use with ultraslim towels that contain superabsorbent polymers. Fertil Steril. 2014;101:515–522.e5.
29. Larsen L, Coyne K, Chwalisz K. Validation of the menstrual pictogram in women with leiomyomata associated with heavy menstrual bleeding. Reprod Sci. 2013;20:680–687.
30. Magnay JL, Nevatte TM, Seitz C, O'Brien S. A new menstrual pictogram for use with feminine products that contain superabsorbent polymers. Fertil Steril. 2013;100:1715–21.e1–4.
31. Wyatt KM, Dimmock PW, Hayes-Gill B, Crowe J, O'Brien PMS. Menstrual symptometrics: A simple computer-aided method to quantify menstrual cycle disorders. Fertil Steril. 2002;78:96–101.
32. Matteson KA, Scott DM, Raker CA, Clark MA. The menstrual bleeding questionnaire: development and validation of a comprehensive patient-reported outcome instrument for heavy menstrual bleeding. Br J Obstet Gynaecol. 2015;122:681–9.
33. Toxqui L, Perez-Granados AM, Blanco-Rojo R, Wright I, Vaquero MP. A simple and feasible questionnaire to estimate menstrual blood loss: relationship with hematological and gynecological parameters in young women. BMC Women's Health. 2014;14 (1):71.
34. Rae C, Furlong W, Horsman J, Pullenayegum E, Demers C, St-Louis J, et al. Bleeding disorders, menorrhagia and iron deficiency: impacts on health-related quality of life. Haemophilia. 2013;19:385–91.
35. Bushnell DM, Martin ML, Moore KA, Richter HE, Rubin A, Patrick DL. Menorrhagia Impact Questionnaire: assessing the influence of heavy menstrual bleeding on quality of life. Curr Med Res Opin. 2010;26:2745–2755.
36. de Souza SS, Camargos AF, Ferreira MC, de Assis Nunes Pereira F, de Rezende CP, Araujo CA, et al. Hemoglobin levels predict quality of life in women with heavy menstrual bleeding. Arch Gynecol Obstet. 2010;281:895–900.
37. Lee LK, Chen PCY, Lee KK, Kaur J. Menstruation among adolescent girls in Malaysia: a cross-sectional school survey. Singapore Med J. 2006;47:869–874.
38. Pawar A, Krishnan R, Davis K, Bosma K, Kulkarni R. Perceptions about quality of life in a school-based population of adolescents with menorrhagia: implications for adolescents with bleeding disorders. Haemophilia. 2008;14:579–83.
39. Warner PE, Critchley HOD, Lumsden MA, Campbell-Brown M, Douglas A, Murray GD. Menorrhagia I: measured blood loss, clinical features, and outcome in women with heavy periods – a survey with follow-up data. Am J Obstet Gynecol. 2004;190:1216–1223.

40. Johannes CB, Crawford SL, Woods J, Goldstein RB, Tran D, Mehrotra S, et al. An electronic menstrual cycle calendar: comparison of data quality with a paper version. Menopause. 2000;7:200–8.
41. Haynes PJ, Hodgson H, Anderson AB, Turnbull AC. Measurement of menstrual blood loss in patients complaining of menorrhagia. Br J Obstet Gynaecol. 1977;84:763–8.
