# Supplementary material for: A systematic review of methods to measure menstrual blood loss
Source: BMC Womens Health. 2018 Aug 22;18:142. doi: 10.1186/s12905-018-0627-8 (PMC6106944; doi:10.1186/s12905-018-0627-8)
Supplement: Supplementary file 5 — Table S5. Correlations of methods with established standards. (PDF 168 kb) [file 12905_2018_627_MOESM5_ESM.pdf]

A systematic review of methods to measure menstrual blood loss

SUPPLEMENTAL TABLE 5

Correlations of methods with established standards.

Author and year (reference)	Method	Study population	N (n)[a]	Correlated variable (reference)	*r*	*P* value	*κ*
**AH**							
Hallberg & Nilsson, 1964 [1]	AH	Healthy women	1 (1)	Iron isotope activity method over three portions of a period	Good agreement	–	–
**Measurement of iron/labelled red blood cells**							
Napolitano et al., 2014 [2]	Menstrual iron loss	Women with normal or confirmed heavy MBL	105 (105)	MFL	0.789	<.001	–
**MFL, duration of menses, number of pads**							
Magnay et al., 2014 [3]	Duration of menses	Women with self-perceived light, normal, or heavy MBL	119 (235)	AH [4]	No correlation<br>No correlation	–	–
	No. of sanitary items			AH [4]	0.97	–	–
	MFL			AH [4]		<.0001	
Schumacher et al., 2012 [5]	MBL estimated from MFL	Women with confirmed heavy MBL	162 (648)	AH [1,6]	0.73	<.0001	–
Gudmundsdottir et al., 2009 [7]	MFL	Women and adolescents with self-perceived normal or heavy MBL, or diagnosed heavy MBL	113 (113)	Subjective MBL	Correlation	–	–
Reid & Virtanen-Kari, 2005 [8]	Total MFL	Women with confirmed heavy MBL (MBL ≥80 mL)	42 (126)	Change in MBL [6]	0.88	<.0001	–
Warner et al., 2004 [9]	Total number of pads/tampons	Women with self-perceived menstrual complaint	226 (225)	AH [1]	0.30	<.001	–
Wyatt et al., 2001 [10]	Number of sanitary items	Women with self-perceived normal or heavy MBL	108 (108)	AH [1]	No overall correlation	–	–
Fraser et al.,	Total log MFL	Women with self-perceived	53 (106)	AH (log MBL) [1,6]	0.93	<.001	–

Author and year (reference)	Method	Study population	N (n)[a]	Correlated variable (reference)	*r*	*P* value	*κ*
2001 [11]		normal or heavy MBL					
Higham & Shaw, 1999 [12]	Duration of menses<br>Number of sanitary items	Women with self-perceived normal or heavy MBL	254 (420)<br>(412)	AH [1]	0.35<br>0.61	<.01<br><.005	–
Vasilenko et al.,1988 [13]	Pad and tampon counts	Women with normal MBL or abnormal MBL	10 (35)	Modified AH method [13]	No correlation	–	–
Fraser et al., 1984 [14]	Pad and tampon counts	Women with self-perceived heavy MBL	60 (240)	AH [1,6]	No relationship	Non-significant	–
Baldwin et al., 1961 [15]	Duration of period	Women with self-perceived normal or heavy MBL	26<br>(67)	MBL determined by Fe^{59}	No relationship	–	–
PBAC							
Zakherah et al., 2011 [16]	PBAC<br>(SAP version)	Women with self-perceived normal or heavy MBL	197 (241)	AH [1,17]	0.60	<.001	–
Reid & Virtanen-Kari, 2005 [8]	PBAC	Women with confirmed heavy MBL (MBL ≥80 mL)	42 (126)	Change in MBL<br>Total MFL	0.53<br>0.58	.0007<br>.0002	–<br>–
Reid et al., 2000 [18]	PBAC	Women with self-perceived heavy MBL	103 (103)	AH [1]	0.4659	–	–
Deeny et al., 1994 [19]	PBAC	Women with dysfunctional uterine bleeding	53 (53)	AH [1]	–	.001	–
Higham et al., 1990 [20]	PBAC	Women with a range of MBL	28 (self-assessed, 55; expert-assessed, 122)	AH [1]	0.847 (self)<br>0.872 (expert)	–<br>–	–<br>–
**Menstrual pictogram**							
Magnay et al., 2014 [3]	Menstrual pictogram (SAP version)	Women with self-perceived light, normal, or heavy MBL	119 (235)	MFL<br>AH [4]	0.85<br>0.81	<.0001<br><.0001	–<br>–
Larsen et al., 2013 [21]	Modified menstrual pictogram (revised icons and excluding extraneous blood)	Women with confirmed heavy menstrual bleeding	87 (87)	Change in AH from baseline [1,6]	0.86 (estimate)	–	0.88 (classification)

Author and year (reference)	Method	Study population	N (n)[a]	Correlated variable (reference)	*r*	*P* value	*κ*
Wyatt et al., 2002 [22]	Menstrual pictogram Symptometrics device	Women with self-perceived normal or heavy MBL	20 (20)	AH [1]	–	<.00001	0.80
Wyatt et al., 2001 [10]	Menstrual pictogram	Women with self-perceived normal or heavy MBL	108 (108)	AH [1]	Good agreement	–	0.8
**Methods involving self-perception of MBL**							
Matteson et al., 2015 [23]	MBQ	Women with self-perceived normal and heavy MBL	67 (–)	SF-36	–0.15 to –0.45	.2 to .0001	–
Toxqui et al., 2014 [24]	MBL score	Women with self-perceived normal MBL	165 (–)	Log ferritin<br>Platelet count	Negative association	.006<br>.011	–<br>–
Bushnell et al., 2010 [25]	MIQ	Women with self-perceived normal MBL or diagnosed heavy MBL	262 (655)	AH	0.3–0.4	<.001	–
de Souza et al., 2010 [26]	SF-36	Women with heavy MBL (>80 mL for ≥3 months)	58 (58)	Physical composite vs PBAC<br>Mental composite vs PBAC<br>Physical composite vs hemoglobin level<br>Mental composite vs hemoglobin level	0.166<br>0.063<br>0.307<br>0.293	.222<br>.642<br>.020<br>.027	–<br>–<br>–<br>–
Pawar et al., 2008 [27]	Quality of life questionnaire	Adolescent women	45 (–)	PBAC	–	<.025	–
Mansfield et al., 2004 [28]	MVJ scale	Women menstruating	31 (89)	Log discharge rate<br>Individual MFL (n = 26)	0.683<br>0.480–0.894	–<br>–	–<br>–
Heath et al., 1999 [29]	Menstrual record<br>Menstrual recall questionnaires	Young adult women	29 (29)	MFL (weighed menstrual loss)	0.47<br>0.61	.012<br>.001	–<br>–

[a]N = study population size; n = number of cycles studied.

AH = alkaline hematin; MBL = menstrual blood loss; MBQ = menstrual bleeding questionnaire; MFL = menstrual fluid loss; MIQ = Menorrhagia Impact Questionnaire; MVJ = Mansfield–Voda–Jorgensen Menstrual Bleeding Scale; PBAC = pictorial blood loss assessment chart; SAP = superabsorbent polymer; SF-36 = Medical Outcomes Study 36-item Short Form Health Survey.

References

1. Hallberg L, Nilsson L. Determination of menstrual blood loss. Scand J Clin Lab Invest. 1964;16:244–248.
2. Napolitano M, Dolce A, Celenza G, Grandone E, Perilli MG, Siragusa S, et al. Iron-dependent erythropoiesis in women with excessive menstrual blood losses and women with normal menses. Ann Hematol. 2014;93:557–63.
3. Magnay JL, Nevatte TM, O'Brien S, Gerlinger C, Seitz C. Validation of a new menstrual pictogram (superabsorbent polymer-c version) for use with ultraslim towels that contain superabsorbent polymers. Fertil Steril. 2014;101:515–522.e5.
4. Magnay JL, Schonicke G, Nevatte TM, O'Brien S, Junge W. Validation of a rapid alkaline hematin technique to measure menstrual blood loss on feminine towels containing superabsorbent polymers. Fertil Steril. 2011;96:394–398.
5. Schumacher U, Schumacher J, Mellinger U, Gerlinger C, Wienke A, Endrikat J. Estimation of menstrual blood loss volume based on menstrual diary and laboratory data. BMC Women's Health. 2012;12 24.
6. Newton J, Barnard G, Collins W. A rapid method for measuring menstrual blood loss using automatic extraction. Contraception. 1977;16:269–282.
7. Gudmundsdottir BR, Hjaltalin EF, Bragadottir G, Hauksson A, Geirsson RT, Onundarson PT. Quantification of menstrual flow by weighing protective pads in women with normal, decreased or increased menstruation. Acta Obstet Gynecol Scand. 2009;88:275–279.
8. Reid PC, Virtanen-Kari S. Randomised comparative trial of the levonorgestrel intrauterine system and mefenamic acid for the treatment of idiopathic menorrhagia: a multiple analysis using total menstrual fluid loss, menstrual blood loss and pictorial blood loss assessment charts. Br J Obstet Gynaecol. 2005;112:1121–5.
9. Warner PE, Critchley HOD, Lumsden MA, Campbell-Brown M, Douglas A, Murray GD. Menorrhagia I: measured blood loss, clinical features, and outcome in women with heavy periods – a survey with follow-up data. Am J Obstet Gynecol. 2004;190:1216–1223.
10. Wyatt KM, Dimmock PW, Walker TJ, O'Brien PMS. Determination of total menstrual blood loss. Fertil Steril. 2001;76:125–131.
11. Fraser IS, Warner P, Marantos PA. Estimating menstrual blood loss in women with normal and excessive menstrual fluid volume. Obstet Gynecol. 2001;98:806–814.
12. Higham JM, Shaw RW. Clinical associations with objective menstrual blood volume. Eur J Obstet Gynecol Reprod Biol. 1999;82:73–76.
13. Vasilenko P, Kraicer PF, Kaplan R, deMasi A, Freed N. A new and simple method of measuring menstrual blood loss. J Reprod Med Obstet Gynecol. 1988;33:293–297.
14. Fraser IS, McCarron G, Markham R. A preliminary study of factors influencing perception of menstrual blood loss volume. Am J Obstet Gynecol. 1984;149:788–93.
15. Baldwin RM, Whalley PJ, Pritchard JA. Measurements of menstrual blood loss. Am J Obstet Gynecol. 1961;81:739–742.
16. Zakherah MS, Sayed GH, El-Nashar SA, Shaaban MM. Pictorial blood loss assessment chart in the evaluation of heavy menstrual bleeding: diagnostic accuracy compared to alkaline hematin. Gynecol Obstet Invest. 2011;71:281–284.
17. Shaw ST, Jr., Aaronson DE, Moyer DL. Quantitation of menstrual blood loss–further evaluation of the alkaline hematin method. Contraception. 1972;5:497–513.
18. Reid PC, Coker A, Coltart R. Assessment of menstrual blood loss using a pictorial chart: a validation study. Br J Obstet Gynaecol. 2000;107:320–322.
19. Deeny M, Davis JA. Assessment of menstrual blood loss in women referred for endometrial ablation. Eur J Obstet Gynecol Reprod Biol. 1994;57:179–180.
20. Higham JM, O'Brien PMS, Shaw RW. Assessment of menstrual blood loss using a pictorial chart. Br J Obstet Gynaecol. 1990;97:734–739.

21. Larsen L, Coyne K, Chwalisz K. Validation of the menstrual pictogram in women with leiomyomata associated with heavy menstrual bleeding. Reprod Sci. 2013;20:680–687.
22. Wyatt KM, Dimmock PW, Hayes-Gill B, Crowe J, O'Brien PMS. Menstrual symptometrics: A simple computer-aided method to quantify menstrual cycle disorders. Fertil Steril. 2002;78:96–101.
23. Matteson KA, Scott DM, Raker CA, Clark MA. The menstrual bleeding questionnaire: development and validation of a comprehensive patient-reported outcome instrument for heavy menstrual bleeding. Br J Obstet Gynaecol. 2015;122:681–9.
24. Toxqui L, Perez-Granados AM, Blanco-Rojo R, Wright I, Vaquero MP. A simple and feasible questionnaire to estimate menstrual blood loss: relationship with hematological and gynecological parameters in young women. BMC Women's Health. 2014;14 (1):71.
25. Bushnell DM, Martin ML, Moore KA, Richter HE, Rubin A, Patrick DL. Menorrhagia Impact Questionnaire: assessing the influence of heavy menstrual bleeding on quality of life. Curr Med Res Opin. 2010;26:2745–2755.
26. de Souza SS, Camargos AF, Ferreira MC, de Assis Nunes Pereira F, de Rezende CP, Araujo CA, et al. Hemoglobin levels predict quality of life in women with heavy menstrual bleeding. Arch Gynecol Obstet. 2010;281:895–900.
27. Pawar A, Krishnan R, Davis K, Bosma K, Kulkarni R. Perceptions about quality of life in a school-based population of adolescents with menorrhagia: implications for adolescents with bleeding disorders. Haemophilia. 2008;14:579–83.
28. Mansfield PK, Voda A, Allison G. Validating a pencil-and-paper measure of perimenopausal menstrual blood loss. Women's Health Issues. 2004;14:242–247.
29. Heath ALM, Skeaff CM, Gibson RS. Validation of a questionnaire method for estimating extent of menstrual blood loss in young adult women. J Trace Elem Med Biol. 1999;12:231–235.
