# Supplementary material for: A systematic review of methods to measure menstrual blood loss
Source: BMC Womens Health. 2018 Aug 22;18:142. doi: 10.1186/s12905-018-0627-8 (PMC6106944; doi:10.1186/s12905-018-0627-8)
Supplement: Supplementary file 6 — Table S6. Assessment of discriminatory power of methods for assessing MBL. (PDF 148 kb) [file 12905_2018_627_MOESM6_ESM.pdf]

A systematic review of methods to measure menstrual blood loss

SUPPLEMENTAL TABLE 6

Assessment of discriminatory power of methods for assessing MBL.

Author(s) and year (reference)	Method	Study population	N (n)[a]	Discriminatory power
**AH**				
Shaw et al., 1972 [1]	Modified AH	Women with self-perceived normal, or abnormal MBL	6 (15)	MBL range: history of HMB, 53.4–728.4 mL; self-perceived normal, 10.6–63.0 mL
van Eijkeren et al., 1986 [2]	Modified AH	Women with self-perceived heavy MBL	21 (21)	Of 21 women who had self-perceived HMB, 24% had measured MBL <80 mL<br>In the remaining 16 women, the MBL range was 80.9–320.9 mL (mean, 154.3 mL)
Vasilenko et al., 1988 [3]	Modified AH	Women with normal or abnormal MBL	10 (35)	MBL range: diagnosed HMB, 90.3−797 mL; normal, 16.0−65.7 mL (mean, 41.8)
Gannon et al., 1996 [4]	Modified AH	Women with heavy MBL	25 (25)	In women with measured MBL <80 mL, self-perceived MBL was: very heavy, 0; heavy, 8 (32%); normal, 8 (32%); light, 3 (20%); very light, 1 (6%); ceased, 5 (20%)
**MFL**				
Gudmundsdottir et al., 2009 [5]	MFL	Women/adolescents with diagnosed heavy MBL or normal MBL	78 (78)	Mean MFL: diagnosed HMB, 217 mL; normal, 51 mL; *P*<.0001
Reid & Virtanen-Kari, 2005 [6]	MFL	Women with confirmed heavy MBL	42 (42)	Median MBL after 6 cycles: levonorgestrel intrauterine system group, 5 mL; mefenamic acid group, 100 mL; *P*<.001<br>Median MFL after 6 cycles: levonorgestrel intrauterine system group, 27 mL; mefenamic acid group, 157 mL; *P*<.001
**Measurement of iron/labelled red blood cells**				
Price et al., 1964 [7]	Fe^{59} whole body counting	Menstruating women	7 (2–4 per patient)	Fe^{59} incorporation into red cell mass: HMB, almost 100%; normal, 19.6%<br>Fe^{59} activity-estimated MBL: history of HMB, 110–550 mL; normal, 33–59 mL
Tauxe, 1962 [8]	Radioactivity counting dome	Menstruating women	22 (NR)	MBL range: mild anemia, 103−341 mL; no anemia, 6−50 mL
Baldwin et al. 1961 [9]	Fe^{59} radioactive iron	Women with self-perceived normal or heavy MBL	26 (67)	Average (range) MBL: self-perceived heavy MBL, 126 mL (50–312 mL); self-perceived normal MBL, 25 mL (10–55 mL)
**PBAC**				

Author(s) and year (reference)	Method	Study population	N (n)[a]	Discriminatory power
Goshtasebi et al., 2015 [10]	Modified PBAC	Women with HMB	76 (152)	Mean PBAC score reduction with three treatment cycles and statistically significant improvements in SF-36 and menorrhagia questionnaire results: 161.31; *P*<.001
Hald & Lieng, 2014 [11]	Modified PBAC	Women with self-perceived light, normal, or heavy MBL	429 (1,049)	Predictive value of diagnosing subjective HMB with a PBAC score cut-off of 150: positive, 61.2%; negative, 87.5%<br>Median PBAC score: subjective heavy MBL, 254.5; normal, 116.0; light, 45.0; *P*<.001
			67	PBAC score reduction with subjective MBL reduction of heavy to normal, 132; *P*<.001<br>PBAC score reduction with subjective MBL reduction of heavy to minimal, 254; *P*<.001
			22	PBAC score difference with subjectively unchanged MBL, 51; *P*=.168
Sanchez et al., 2012 [12]	PBAC	Menstruating adolescents	73 (73)	Mean PBAC score: self-perceived heavy MBL, 362; normal, 136; light, 44; *P*<.002<br>Of 42 women identifying as having "normal" menses, >60% had a PBAC score >100
Zakherah et al., 2011 [13]	PBAC	Women with self-perceived normal or heavy MBL	197 (241)	PBAC score was an independent predictor of MBL >80 mL (adjusted odds ratio, 10.1; area under the receiver operator characteristics curve, 0.819)<br>Average PBAC score: subjective HMB, 225; normal, 120
Nahidi et al., 2011[14]	Modified PBAC	Women with or without menstrual disorder	160 (160)	There were significant differences between women with and without menstrual disorder in the number of products used and the amount of bleeding (both *P*=.009)
Biri et al., 2007 [15]	PBAC	Menstruating women	600 (600)	Women using five or more pads during menstruation who had a PBAC score >50, 79%; women having blood on their underwear who had a PBAC score >50, 67%; women using large-sized pads who had a PBAC score >50, 76%
Reid & Virtanen-Kari, 2005[6]	PBAC	Women with confirmed heavy MBL	42 (42)	Median MBL after six cycles: levonorgestrel intrauterine system group, 5 mL; mefenamic acid group, 100 mL; *P*<.001<br>Median PBAC score after six cycles: levonorgestrel intrauterine system group, 25; mefenamic acid group, 159; *P*<.001
Reid et al., 2000 [16]	PBAC	Women with self-perceived heavy MBL	103 (103)	Predictive value of diagnosing HMB with a PBAC score cut-off of 100: positive, 62%; negative, 60%
Janssen et al., 1995 [17]	Modified PBAC<br>Subjective assessment	Women with self-perceived normal or heavy MBL or unexplained anemia	288 (489)	Predictive value of diagnosing HMB with a PBAC score cut-off of 185: positive, 85.9%; negative, 84.8%<br>Predictive value of diagnosing HMB with a complaint of heavy MBL: positive, 55.9%
**Menstrual pictogram**				
Larsen et al., 2013 [18]	Menstrual pictogram	Women with confirmed heavy MBL	170 (169)	Predictive value of diagnosing HMB: positive, 91%; negative, 83%
Wyatt et al., 2001 [19]	Menstrual pictogram	Women with self-perceived normal or heavy MBL	108 (108)	Median menstrual pictogram MBL: self-perceived HMB, 67 mL; normal, 31 mL

Author(s) and year (reference)	Method	Study population	N (n)[a]	Discriminatory power
**Methods involving self-perception of MBL**				
Matteson et al., 2015 [20]	MBQ	Women with self-perceived heavy or normal MBL	144 (NR)	Mean MBQ score: self-perceived HMB, 10.6; normal, 30.8; *P*<.0001
Karlsson et al., 2014 [21]	Self-perception of MBL	Women with self-perceived heavy or normal MBL	1483 (NR)	Bleeding taken into account when planning activities: self-perceived HMB, 25%; normal, 7%; *P*<.0001<br>Refrained from activities because of bleeding: HMB, 22%; normal, 2%; *P*<.0001<br>Absent from work 1–5 days a year owing to MBL: HMB, 14%; normal, 2%; *P*<.0001
Magnay et al., 2014 [22]	Self-perception of MBL	Women with self-perceived light, normal, or heavy MBL	53 (NR)	Of the 53 women who had self-perceived HMB, 26% had MBL >80 mL
Toxqui et al., 2014 [23]	MBL score	Healthy women	165 (NR)	Mean MBL score: high MBL by cluster analysis, 6.0; low MBL by cluster analysis, 2.4
Rae et al., 2013 [24]	Health utilities index	Women with von Willebrand disease and other bleeding disorders	185 (185)	Mean health-related QoL: HMB diagnosed by Clinical History Assessment Tool, 0.64; without HMB, 0.82; *P*<.001<br>Percentage of women for whom menstrual bleeding interfered with daily activities: HMB, 77.5%; without HMB, 42.7%
Schumacher et al., 2012 [25]	Mixed linear model (diary and laboratory parameters)	Women with confirmed heavy MBL (≥80 mL during at least two reference cycles)	162 (648)	Mean (geometric mean/median) estimated MBL value vs the measured value: heavy-bleeding days, 70.25 mL (67.44/68.42 mL) vs 60.86 mL (48.01/51.20 mL); normal-bleeding days, 31.57 mL (29.36/30.05 mL) vs 30.39 mL (18.21/22.10 mL); light-bleeding days, 7.52 mL (6.36/7.27 mL) vs 9.23 mL (4.70/4.00 mL); spotting days, 3.23 mL (2.19/3.16 mL) vs 3.04 mL (1.65/1.10 mL)
Bushnell et al., 2010 [26]	MIQ	Women with self-perceived normal MBL or diagnosed heavy MBL	262 (524)	For each MIQ item, the mean baseline score for the treatment group was much higher than the mean score in the control group; *P*<.001<br>For the treatment group, significant differences were detected for changes in MIQ items 1–4 from baseline to month 1; *P*<.001
Pawar et al., 2008 [27]	QoL questionnaire	Adolescent women	45 (NR)	Perceived their period as heavy: PBAC score ≥100, 22/ 25 (88%); *P*<.001; PBAC score <100, 10/20 (50%); *P*<.99<br>Missed school during their menstrual periods: PBAC score ≥100, 13/25 (52%); PBAC score <100, 4/20 (20%); *P*<.07
Mansfield et al. 2004 [28]	MVJ	Menstruating women	31 (89)	All MVJ categories were statistically unique except categories 1 and 2<br>Mean MBL: MVJ score of 6, 107.7 mL; MVJ score of 5 or 6, 67.2 mL
Warner et al., 2004 [29]	MEQ	Women with self-perceived heavy MBL	226 (225)	Mean measured MBL: ‘‘very heavy’’, 64 mL; other, 40 mL; *P*<.001<br>Of 226 women who had self-perceived HMB, 34% had MBL >80 mL
Wyatt et al., 2001 [19]	Self-perception of MBL	Women with self-perceived heavy MBL	61 (61)	Of the 61 women who had self-perceived HMB, 36% had MBL >80 mL (if extraneous loss was included, 74%)

Author(s) and year (reference)	Method	Study population	N (n)[a]	Discriminatory power
Reid et al., 2000 [16]	Self-perception of MBL	Women with self-perceived heavy MBL	103 (103)	Of the 103 women with self-perceived HMB, 61.1% had MBL >80 mL
Higham & Shaw, 1999 [30]	Clinical parameters	Women with self-perceived normal or heavy MBL	254 (254)	Median MBL: HMB, 79 mL (≥80 mL, 49%; ≥60 ml, 66%); controls, 36 mL (≥80 mL, 27.7%)
Heath et al., 1999 [31]	Menstrual Record<br>Menstrual Recall	Young adult women	29 (29)	Reference weighed loss (MFL) was significantly different between the surrogate categories of second and third tertiles for the Record method, and between the first and third tertiles for the Recall method; *P*≤.05
Fraser et al., 1984 [32]	Self-perception of MBL<br><br>Subjective daily assessment<br>Duration of menstruation	Women with a self-complaint and convincing history of heavy MBL	69 included; 60 with perception data (240)	Of the 69 patients with self-perceived HMB, 41 (59%) had measured MBL >80 mL in either or both of two cycles<br>MBL: in the "lightest" cycles was less than in the "heaviest" cycles; *t* = 3.098; *P*<.001); however, only 45% of 60 women correctly selected the order of all four periods from lightest to heaviest<br>The difference in MBL between the four points on the daily rating scale was highly significant; however, the range in each category was very wide: +++, 1.4–215.8 mL; ++, 0.5–108.6 mL; +, 0.1–63.1 mL; 0, 0.1–15.5 mL<br>Duration of bleeding: lightest periods, 4.7 ± 0.4 days; heaviest periods, 5.8 ± 0.8 days
Chimbira et al., 1980 [33]	Self-perception of MBL	Women with self-perceived heavy, medium and light MBL	92 (184)	Of the patients with self-perceived light MBL, 34% had measured MBL >80 mL (median, 63 mL); of those with self-perceived medium MBL, 55% had measured MBL >80 mL (median, 99 mL); of those with self-perceived HMB, 47% had measured MBL <80 mL (median, 97 mL)
Haynes et al., 1977 [34]	Self-perception<br>Duration of menstruation	Women with self-perceived heavy MBL	66 (132)<br><br>50 (100)	53% of women self-diagnosed with HMB repeatedly had measured MBL >80 mL<br>76% of women self-diagnosed with HMB had ≥1cycle with measured MBL >80 mL<br>There was no relationship between the duration of menstruation and total MBL: only 45% of women who bled for more than 7 days had a measured MBL >80 mL

[a]N, study population size; n, number of cycles studied.

AH = alkaline hematin; HMB = heavy menstrual bleeding; MBL = menstrual blood loss; MBQ = Menstrual Bleeding Questionnaire; MEQ = Menstrual Evaluation Questionnaire; MFL = menstrual fluid loss; MIQ = Menorrhagia Impact Questionnaire; MVJ = Mansfield–Voda–Jorgensen Menstrual Bleeding Scale; NR = not reported; PBAC = pictorial blood loss assessment chart; QoL = quality of life; SF-36 = Medical Outcomes Study 36-item Short-Form Health Survey.

References

1. Shaw ST, Jr., Aaronson DE, Moyer DL. Quantitation of menstrual blood loss–further evaluation of the alkaline hematin method. Contraception. 1972;5:497–513.
2. van Eijkeren MA, Scholten PC, Christiaens GC, Alsbach GP, Haspels AA. The alkaline hematin method for measuring menstrual blood loss–a modification and its clinical use in menorrhagia. Eur J Obstet Gynecol Reprod Biol. 1986;22:345–51.
3. Vasilenko P, Kraicer PF, Kaplan R, deMasi A, Freed N. A new and simple method of measuring menstrual blood loss. J Reprod Med Obstet Gynecol. 1988;33:293–297.
4. Gannon MJ, Day P, Hammadieh N, Johnson N. A new method for measuring menstrual blood loss and its use in screening women before endometrial ablation. Br J Obstet Gynaecol. 1996;103:1029–1033.
5. Gudmundsdottir BR, Hjaltalin EF, Bragadottir G, Hauksson A, Geirsson RT, Onundarson PT. Quantification of menstrual flow by weighing protective pads in women with normal, decreased or increased menstruation. Acta Obstet Gynecol Scand. 2009;88:275–279.
6. Reid PC, Virtanen-Kari S. Randomised comparative trial of the levonorgestrel intrauterine system and mefenamic acid for the treatment of idiopathic menorrhagia: a multiple analysis using total menstrual fluid loss, menstrual blood loss and pictorial blood loss assessment charts. Br J Obstet Gynaecol. 2005;112:1121–5.
7. Price DC, Forsyth EM, Cohn SH, Cronkite EP. The study of menstrual and other blood loss, and consequent iron deficiency, by Fe^{59} whole-body counting. Can Med Assoc J. 1964;90:51–4.
8. Tauxe WN. Quantitation of menstrual blood loss: a radioactive method utilizing a counting dome. J Nucl Med. 1962;3:282–287.
9. Baldwin RM, Whalley PJ, Pritchard JA. Measurements of menstrual blood loss. Am J Obstet Gynecol. 1961;81:739–742.
10. Goshtasebi A, Mazari Z, Behboudi Gandevani S, Naseri M. Anti-hemorrhagic activity of Punica granatum L. flower (Persian Golnar) against heavy menstrual bleeding of endometrial origin: a double-blind, randomized controlled trial. Med J Islam Repub Iran. 2015;29:199.
11. Hald K, Lieng M. Assessment of periodic blood loss: interindividual and intraindividual variations of pictorial blood loss assessment chart registrations. J Min Invas Gynecol. 2014;21:662–668.
12. Sanchez J, Andrabi S, Bercaw JL, Dietrich JE. Quantifying the PBAC in a pediatric and adolescent gynecology population. Pediatr Hematol Oncol. 2012;29:479–484.
13. Zakherah MS, Sayed GH, El-Nashar SA, Shaaban MM. Pictorial blood loss assessment chart in the evaluation of heavy menstrual bleeding: diagnostic accuracy compared to alkaline hematin. Gynecol Obstet Invest. 2011;71:281–284.
14. Nahidi F, Bagheri L, Jannesari S, Majd HA. Relationship between delivery type and menstrual disorders: a case–control study. J Res Health Sci. 2011;11:83–90.
15. Biri A, Bozkurt N, Korucuoglu U, Yilmaz E, Tiras B, Guner H. Use of pictorial chart for managing menorrhagia among Turkish women. J Turk Ger Gynecol Assoc. 2007;9:35–37.
16. Reid PC, Coker A, Coltart R. Assessment of menstrual blood loss using a pictorial chart: a validation study. Br J Obstet Gynaecol. 2000;107:320–322.
17. Janssen CAH, Scholten PC, Heintz APM. A simple visual assessment technique to discriminate between menorrhagia and normal menstrual blood loss. Obstet Gynecol. 1995;85:977–982.
18. Larsen L, Coyne K, Chwalisz K. Validation of the menstrual pictogram in women with leiomyomata associated with heavy menstrual bleeding. Reprod Sci. 2013;20:680–687.
19. Wyatt KM, Dimmock PW, Walker TJ, O'Brien PMS. Determination of total menstrual blood loss. Fertil Steril. 2001;76:125–131.

20. Matteson KA, Scott DM, Raker CA, Clark MA. The menstrual bleeding questionnaire: development and validation of a comprehensive patient-reported outcome instrument for heavy menstrual bleeding. Br J Obstet Gynaecol. 2015;122:681–9.
21. Karlsson TS, Marions LB, Edlund MG. Heavy menstrual bleeding significantly affects quality of life. Acta Obstet Gynecol Scand. 2014;93:52–7.
22. Magnay JL, Nevatte TM, O'Brien S, Gerlinger C, Seitz C. Validation of a new menstrual pictogram (superabsorbent polymer-c version) for use with ultraslim towels that contain superabsorbent polymers. Fertil Steril. 2014;101:515–522.e5.
23. Toxqui L, Perez-Granados AM, Blanco-Rojo R, Wright I, Vaquero MP. A simple and feasible questionnaire to estimate menstrual blood loss: relationship with hematological and gynecological parameters in young women. BMC Women's Health. 2014;14 (1):71.
24. Rae C, Furlong W, Horsman J, Pullenayegum E, Demers C, St-Louis J, et al. Bleeding disorders, menorrhagia and iron deficiency: impacts on health-related quality of life. Haemophilia. 2013;19:385–91.
25. Schumacher U, Schumacher J, Mellinger U, Gerlinger C, Wienke A, Endrikat J. Estimation of menstrual blood loss volume based on menstrual diary and laboratory data. BMC Women's Health. 2012;12 24.
26. Bushnell DM, Martin ML, Moore KA, Richter HE, Rubin A, Patrick DL. Menorrhagia Impact Questionnaire: assessing the influence of heavy menstrual bleeding on quality of life. Curr Med Res Opin. 2010;26:2745–2755.
27. Pawar A, Krishnan R, Davis K, Bosma K, Kulkarni R. Perceptions about quality of life in a school-based population of adolescents with menorrhagia: implications for adolescents with bleeding disorders. Haemophilia. 2008;14:579–83.
28. Mansfield PK, Voda A, Allison G. Validating a pencil-and-paper measure of perimenopausal menstrual blood loss. Women's Health Issues. 2004;14:242–247.
29. Warner PE, Critchley HOD, Lumsden MA, Campbell-Brown M, Douglas A, Murray GD. Menorrhagia I: measured blood loss, clinical features, and outcome in women with heavy periods – a survey with follow-up data. Am J Obstet Gynecol. 2004;190:1216–1223.
30. Higham JM, Shaw RW. Clinical associations with objective menstrual blood volume. Eur J Obstet Gynecol Reprod Biol. 1999;82:73–76.
31. Heath ALM, Skeaff CM, Gibson RS. Validation of a questionnaire method for estimating extent of menstrual blood loss in young adult women. J Trace Elem Med Biol. 1999;12:231–235.
32. Fraser IS, McCarron G, Markham R. A preliminary study of factors influencing perception of menstrual blood loss volume. Am J Obstet Gynecol. 1984;149:788–93.
33. Chimbira TH, Anderson ABM, Turnbull AC. Relation between measured menstrual blood loss and patient's subjective assessment of loss, duration of bleeding, number of sanitary towels used, uterine weight and endometrial surface area. Br J Obstet Gynaecol. 1980;87: 603–9.
34. Haynes PJ, Hodgson H, Anderson AB, Turnbull AC. Measurement of menstrual blood loss in patients complaining of menorrhagia. Br J Obstet Gynaecol. 1977;84:763–8.
